# Rhodotorula mucilaginosa ZTHY2 as a promising antibiotic alternative in Leizhou black ducks: impact on growth, meat quality, intestinal health, and microbiota composition

**DOI:** 10.1186/s12917-026-05292-3

**Published:** 2026-01-30

**Authors:** Jiang Wu, Namula Zhao, Wei Yang, Yingxin Hu, Zhibao Chen

**Affiliations:** 1 Department of Veterinary Medicine, College of Coastal Agricultural Sciences, Guangdong Ocean University, Zhanjiang, 524088 China; 2 College of Veterinary Medicine, South China Agricultural University, Guangzhou, 510642 China; 3 South China Branch of National Saline-Alkali Tolerant Rice Technology Innovation Center Zhanjiang, Zhanjiang, 524088 China

**Keywords:** Rhodotorula mucilaginosa ZTHY2, Leizhou black duck, Growth performance, Meat quality, Intestinal health

## Abstract

Rhodotorula mucilaginosa ZTHY2 is safe, and can produce abundant nutrient metabolites, supporting the “no-resistance” culture as an additive in animal feed. In this study, the Leizhou black duck was chosen as the model organism to investigate the effects of ZTHY2 on animal growth, meat quality, intestinal health, and the gut microbiota composition. A total of 150 healthy, 1-day-old male ducks were randomly divided into five groups with 3 replicates each, with 10 birds per replicate. The control group was fed a standard diet, whereas the RM group received a diet supplemented with 2 × 10^7^, 2 × 10^8^, or 2 × 10^9^ CFU/kg ZTHY2. The LA group was fed a diet supplemented with 2 × 10^9^ CFU/kg Lactobacillus acidophilus. The experiment lasted 42 days. The analysis revealed significant improvements in the average daily gain for both the RM and LA groups over the control, with *P* < 0.05. The feed conversion ratios also significantly decreased at RM dosages of 2 × 10^8^ CFU/kg and 2 × 10^9^ CFU/kg, with *P* < 0.05. Notably, 2 × 10^9^ CFU/kg RM improved the carcass yield percentage, reduced muscle water loss, and significantly altered the meat color L* and b* values in the ducks (*P* < 0.05). Additionally, it increased the a* value of pectoral muscles and the pH_24h_ value (*P* < 0.05). The crude protein content of the pectoral muscle was significantly greater (*P* < 0.05) in the 2 × 10^9^ CFU/kg RM and 2 × 10^9^ CFU/kg LA groups, whereas the crude fat content was lower in the 2 × 10^7^ CFU/kg, 2 × 10^9^ CFU/kg RM, and 2 × 10^9^ CFU/kg LA groups (*P* < 0.05) relative to the control. ZTHY2 administration significantly modified the amino acid and fatty acid profiles in duck muscles, increasing the contents of threonine and tyrosine and decreasing the proportion of nervonic acid (*P* < 0.05). ZTHY2 significantly increased the villus height and reduced crypt depth across all intestinal segments (*P* < 0.05), indicating improved morphology and architectural integrity. Additionally, ZTHY2 stabilized the intestinal microbiota, significantly improved the composition and structure of the gut floral community. In summary, the results suggested that ZTHY2 could improve the growth performance and muscle texture of the ducks by regulating intestinal microflora, promote intestinal health, and enhance growth and muscle quality. The most notable improvements were observed at 2 × 10^9^ CFU/kg ZTHY2. This study provided a theoretical basis for incorporating Rhodotorula mucilaginosa ZTHY2 into nonmedicated poultry feed formulations.

**Supplementary Information:**

The online version contains supplementary material available at 10.1186/s12917-026-05292-3.

## Introduction

To address the escalating issue of antibiotic resistance, the European Union has implemented a regulatory policy that prohibits the use of antibiotics as dietary supplements in feed for livestock and poultry, which had been effective since January 1, 2006 [1]. In light of the European Union’s successful ban, which has yielded positive results and extensive experience, China implemented relevant bans and limits in 2020 to control the use of growth-promoting antibiotics in animal husbandry. Consequently, the feed additive market was shifting toward more environmentally friendly, secure, and residue-free options [2, 3]. Additionally, feed additives that increase animal nutritional value, promote animal health, increase breeding efficiency, and improve the quality of livestock and poultry products are becoming popular [4–6]. This trend had led to a growing focus on probiotic-based microbial preparations [7], such as those containing Saccharomyces cerevisiae, in the research for alternative feed additives [8].

Rhodotorrhoea colulosa was classified as a microorganism with intermediate temperature tolerance, with an optimal growth range of 28 °C to 30 °C. Therefore, a diverse nutrient profile was required for its propagation, characterized by a reduced culture cost and a shortened growth cycle [9]. Certain strains of Rhodosaccharomyces presented a relatively high content of polyunsaturated fatty acids, enabling them to synthesize and amass significant quantities of oil within their cells, positioning them as natural candidates for microbial oil production [10]. Rhodosaccharomyces colloides was abundant in proteins, minerals, amino acids, polysaccharides, polyunsaturated fatty acids, and other nutritive components but also contained high amounts of carotenoids, beta-glucan, digestive enzymes, B vitamins, and other bioactive metabolites [11]. Due to these nutrients, Rhodotorrhoea have diverse biological benefits, for example, it could boost animal immunity, bolster antioxidant defenses, foster growth and development, improve the quality of livestock products, and support intestinal health, positioning them as outstanding candidates for novel natural feed additives [3, 8]. Akiba et al. developed a marine red yeast additive with a biomass of 120 µg/g via spray drying technology, this yeast was used as a feed additive for laying hens to increase fat content, increase egg weight, improve growth rates, and increase yolk color, laying rates, and egg quality while reducing laying hen mortality [12]. Furthermore, the addition of Rhodotorula mucilaginosa, a yeast isolated from soil enriched with citrus and grape waste, has been demonstrated to improve growth, increase antioxidant activity, enhance gastrointestinal digestion, and maintain the intestinal microbial balance in piglets [13]. The above research shows that the abundant nutrients in S. rubrosacchari benefit animal growth and improve product quality, providing significant economic advantages for livestock, poultry, and aquaculture.

Rhodosaccharomyces colloides is a transient bacterium that traverses the animal intestinal tract in a viable state without adhering to the intestinal mucosa [3]. The β-glucan present in the cell wall of this yeast has been shown to increase the villous height and crypt depth in the intestine, thereby increasing the contact area of the intestine with dietary nutrients, improving feed absorption and utilization, and ultimately promoting animal growth and development [14]. Upon entering the animal gastrointestinal tract, Rhodoceras coleus created an anaerobic environment by consuming oxygen, promoting competition between beneficial bacteria such as Bifidobacterium and Lactobacillus and harmful bacteria for colonization sites [13]. Rhodoceras coleus had been demonstrated to exert a beneficial influence on the intestinal architecture, preserving equilibrium in the gut microbiota and reinforcing intestinal barrier functions, thereby underpinning intestinal health in animals [15–17]. Study had been shown that adding 10 g/kg Saccharomyces cerevisiae to the diet of weanling pigs daily for 28 days significantly improved their growth rate, reduced the feed-to-gain ratio, and increased the digestibility of nutrients [18]. Saccharomyces cerevisiae and its fermentation products significantly benefited animal husbandry by improving growth, overall health, and egg production in livestock and poultry [3, 18]. As a natural supplement, Saccharomyces cerevisiae was promising for sustainable animal agriculture and production economics. However, its effects as a feed additive on growth, carcass yield, and intestinal integrity in meat ducks had yet to be investigated, and the optimal dosage avian species was undetermined.

The Leizhou black duck has a rich history of breeding and has flourished in the secluded coastal beach environment of tropical and subtropical zones. It represents a valuable germplasm resource within the livestock and poultry population of the Leizhou Peninsula [3, 19]. Rhodotorula mucilaginosa ZTHY2, a unicellular eukaryotic microorganism of the marine Rhodiomyces genus, was selected for this study and was previously isolated and purified from the coastal waters of the Leizhou Peninsula by our research team [3, 8]. This strain has a short growth cycle, a high number of somatic cells, and low nutritional requirements. Additionally, a pathogenicity test in mice confirmed that this strain is nontoxic, safe, and reliable [3]. Specifically, although probiotic research in poultry is extensive, most investigations have focused on the genera Saccharomyces and Lactobacillus; systematic information on the mode of action, dose–response relationship, and combined effects of Rhodotorula mucilaginosa on meat quality and the gut microbiome of poultry is still lacking. Moreover, this is the first study to use the local Leizhou black-duck breed as a model to evaluate the comprehensive benefits of strain ZTHY2 under antibiotic-free conditions, thereby filling a knowledge gap regarding the application of this yeast in indigenous waterfowl.

We hypothesized that dietary ZTHY2, by delivering bioactive metabolites, enhances intestinal villus–crypt architecture and muscle protein accretion through modulation of lipid-associated nuclear signaling and mucosal barrier tightening. Therefore, in this study, we investigated the optimal dosage of ZTHY2 as a feed additive substitute by examining its effects on the growth, slaughter, immune function, and intestinal health of the Leizhou black duck. The results provide a scientific basis for the application of Rhodotorula mucilaginosa ZTHY2 in avian breeding, enhancing the safety and quality of livestock products and promoting regional agricultural development.

## Materials and methods

### Experimental strains

Rhodotorula mucilaginosa ZTHY2, a carotenoid-producing yeast, was isolated from the marine environment of the Leizhou Peninsula and deposited at the China Center for Type Culture Collection under accession number M2015296. Lactobacillus acidophilus (BNCC185342) was obtained from Beina Biotechnology Co., Ltd.

### Experimental animals and group design

A total of 150 healthy, similarly sized, 1-day-old male Leizhou black ducks (Leizhou Feihuang Animal Husbandry Breeding Co., Ltd.) were selected and randomly assigned to five treatment groups, each with three replicates and ten birds per replicate. The ducks in the control group were provided a standard basal diet, whereas those in the RM groups received a basal diet supplemented with 2 × 10^7^ CFU/kg, 2 × 10^8^ CFU/kg, or 2 × 10^9^ CFU/kg of Rhodotorula mucilaginosa ZTHY2; these groups were referred to as Control (Control group), RM1 (2 × 10^7^ CFU/kg RM group), RM2 (2 × 10^8^ CFU/kg RM group), and RM3 (2 × 10^9^ CFU/kg RM group), respectively. The LA group was fed a diet supplemented with 2 × 10^9^ CFU/kg Lactobacillus acidophilus, referred to as the LA group. The feeding trial lasted 42 days.

Forty-two-day-old Leizhou black ducks were euthanized in strict accordance with the Guidelines for Euthanasia of Laboratory Animals (Laboratory Animal Center, Peking University, 2021) and the GB/T 39,760 − 2021 standard. Sodium pentobarbital (≥ 150 mg/kg) was administered intravenously to induce deep anesthesia; after the corneal reflex was abolished and muscle tone was completely lost, exsanguination via the jugular vein was performed immediately until cardiac and respiratory arrest were confirmed and pupillary dilation was maintained for ≥ 2 min. Throughout the procedure, the birds were protected from pain and stress.

### Feeding management and basic feed

Prior to the trial, the duck housing and utensils underwent a complete cleaning and disinfection process. After proper ventilation and purification, the experiment began. It employed an indoor, horizontal feeding system with a varied diet. The Leizhou black ducks had continuous access to feed and water, while the facility’s temperature, humidity, and lighting were carefully controlled. Regular cleaning maintained good ventilation. The ducks’ health was continually monitored, with a focus on the drinking water quality. Timely feed and water supply were ensured, with detailed records kept. The feeding and vaccination protocols followed standard industry practices. The basal feed met the requirements of the Meat Duck Feeding Standard (NY/T 2122 − 2012), and its composition and nutritional level are detailed in Table 1.

**Table 1:** Dietary composition and nutrient concentrations of basal diets (Air-Dry Basis) in percentages

Items	1–21 days	22–42 days	Items	1–21 days	22–42 days
Ingredients			Nutrition levels		
Corn	61.85	64.60	ME/(MJ/kg)	12.24	12.35
Soybean meal	28.50	25.30	CP	20.70	18.60
Wheat bran	3.25	3.14	Ca	0.98	0.93
Fish meal	2.50	2.80	AP	0.45	0.40
Nacl	0.30	0.30	Lys	1.10	0.86
Limestone	1.30	1.29	Met	0.50	0.45
CaHPO_4_	1.28	1.26			
DL-Met	0.19	0.21			
Premix^1^	1.00	1.10			
Total	100.00	100.00			

^1^The dietary premix supplies the following nutritional components per kilogram of starter: Vitamin A, 8,000 International Units (IU); Vitamin D, 2,000 IU; Vitamin E, 20 IU; Vitamin B12, 12 milligrams (mg); Vitamin K3, 2 mg; nicotinic acid, 40 mg; D-pantothenic acid, 15 mg; folic acid, 1 mg; iodine, 0.40 mg; iron, 60 mg; copper, 8 mg; zinc, 60 mg; manganese, 100 mg; and selenium, 0.35 mg

### Growth performance

Routine weigh-ins and detailed records of feed consumption were documented for the Leizhou black ducks on days 1, 7, 14, 21, 28, 35, and 42 of the trial. Thereafter, comprehensive calculations were conducted to ascertain the mean body weight (BW), average daily feed intake (ADFI), average daily gain (ADG), and feed conversion ratio (FCR).

BW (g) = total weight/number of ducks.

ADFI (g/d) = material consumption during the test period/test days.

ADG (g/d) = (average weight at the end of the trial - average weight at the beginning of the trial)/test days.

FCR = average daily feed intake/average daily gain.

### Animal slaughter efficiency

During the 42-day experimental period, two Leizhou black ducks with similar body weights and health statuses were randomly chosen from each group for preslaughter weighing after a 12-hour fasting period. Subsequently, jugular vein bleeding was performed on these ducks, followed by measurement of their body weight and dissection to isolate and collect the bilateral pectoral and leg muscles. The assessments were conducted in compliance with the agricultural industry standard NY/T 823 − 202 to calculate the slaughter rate, half evisceration rate, full evisceration rate, pectoral muscle rate, leg muscle rate, and lean meat rate.

Slaughter rate (%) = body weight/body weight before slaughter ×100.

Half bore rate (%) = half bore weight/body weight before slaughter ×100.

Full bore rate (%) = full bore weight/body weight before slaughter × 100.

Leg muscle percentage (%) = weight of both leg muscles/full evisceration weight ×100.

Chest muscle percentage (%) = weight of both chest muscles/full evisceration weight ×100.

Lean meat percentage (%) = (weight of the chest muscle from both sides + weight of the leg muscle from both sides)/full bore weight ×100.

### Evaluation of meat quality indicators

The conventional meat quality parameters, including pH, meat color, water loss rate, cooking loss rate, and shear force, were assessed in the left breast and leg muscles of 42-day-old Leizhou black ducks. pH was measured at three points on the carcass using a pH-Star probe (Matthäus, Germany) calibrated at ambient temperature. Surface meat colour (L*a*b*) was recorded with a CR-400 colorimeter (Konica Minolta, Japan; illuminant D65, 10° observer) after white-plate calibration, and values from three locations on the breast muscle were averaged. Water-holding capacity was assessed as drip loss by the gravitative compression method (5 kg, 5 min, 4 °C) employing a TX-5001 bench-top compression device (Xiongchuan, Suzhou, China).

Flesh color: A color meter probe was placed vertically over the muscle cross-section to measure the brightness (L*), saturation (a*), and yellow–blue (b*) indices. Consistent readings were obtained, and the values were recorded and replicated three times for accuracy.

pH45min: Within 45–60 min postmortem, three specific regions of the lateral chest and leg muscles were chosen. An inline meat pH meter probe was then inserted into each sample to determine the pH. Once the pH readings stabilized, the values were recorded and averaged to serve as the final pH value for each muscle area.

pH24h: The meat sample was stored at 4 °C in a refrigerator, and its pH value was determined at 24 h postmortem via the same analytical method as that used at 45 min postmortem.

Water loss rate: A precise circular corer was used to obtain two tissue samples (each with a cross-sectional area of 2.5 cm² and a thickness of 1 cm) from the pectoral and femoral muscles. These samples were weighted and denoted as M1. Next, the tissue samples were placed on a flat pressure application surface, wrapped in a stack of 16 sheets of qualitative filter paper, and a pressure load of 35 kg was applied for 5 min. After pressure application, the samples were weighed again, and the weight was recorded as M2.

Water loss rate (%) = (M1-M2)/M1 × 100%.

Cook loss: The chest and leg muscles were stored at 4 °C for up to 24 h postslaughter to assess cook loss. The meat was subsequently cubed parallel to the muscle fibers, and the weight was recorded as M3. The sample was then placed in a zip-lock bag and cooked in a water bath at 80 °C until the core temperature reached 75 °C. Upon cooling to room temperature, the meat was removed, the surface moisture was drained with filter paper, and the meat weight was recorded as M4.

Cook loss (%) = (M3-M4)/M3 × 100%.

Shear force: Shear force evaluation was conducted on cooked meat samples via a meat texture analyzer, with each sample being assessed twice to determine the mean value.

### Routine analysis of muscle nutrient composition

Refined moisture, crude ash, crude fat, and crude protein contents were determined in the pectoral and femoral muscles of a 42-day-old Leizhou black duck following the removal of superficial fat and fascia. The moisture content was assayed via a direct drying method in accordance with the national standard GB/5009.3–2016. The determination of crude fat content was performed via the Soxhlet extraction method as stipulated in the national standard GB/5009.6–2016. Crude protein content was evaluated via the Kjeldahl nitrogen determination method by referencing the national standard GB/5009.5–2016. Crude ash content was assessed following the protocol outlined in the national standard GB/5009.4–2016.

### Analysis of fatty acids and amino acids in muscle tissue

The amino acid and fatty acid profiles of 42-day-old Leizhou black duck muscles were assessed after the meat was homogenized into a paste. The amino acid analysis was based on the “National Food Safety Standard: Determination of Amino Acids in Foods” (GB 5009.124–2016), and an automatic amino acid analyzer was used for quantification, with validation performed through retention time and peak area comparisons with standards. Fatty acid analysis was based on the “National Food Safety Standard: Determination of Fatty Acids in Foods” (GB/T 5009.168–2016). Following sample hydrolysis and fat extraction, fatty acid methyl esters were produced via saponification and methylation. The relative percentage of fatty acid methyl esters was calculated via peak area normalization, which was supported by the determination of the relative retention times of fatty acid methyl ester standards via gas chromatography.

### Intestinal histomorphological analysis

Small intestinal segments 21 and 42 were preserved in 4% paraformaldehyde for more than 72 h. Using surgical blades, we excised these segments, which were approximately 2–3 millimeters long, to ensure parallel and planar terminal ends of the cross-sections. The samples were then prepared for embedding and placed in a molding box. After embedding, the wax blocks were cut into 5-µm-thick sections for HE staining. The stained slides were dried, hardened, sealed, and finally examined under a microscope. The intestinal villus height (VH) and crypt depth (CD) were measured, and the V/C ratio was calculated.

### Investigation into the composition of intestinal microbiota

On the 42nd day, two Leizhou black ducks with similar body weights were randomly chosen for slaughter from each group. Their cecal contents were aspirated aseptically into 5 ml sterilized centrifuge tubes, quickly frozen in liquid nitrogen, and stored at -80 °C for later analysis. The samples were as follows: control (control group), RM1 (2 × 10^7^ CFU/kg of RM), RM2 (2 × 10^8^ CFU/kg of RM), RM3 (2 × 10^9^ CFU/kg of RM), and LA (2 × 10^9^ CFU/kg LA).

Following the comprehensive extraction of microbiome DNA, target fragment amplification via polymerase chain reaction (PCR), subsequent product recovery and purification, and fluorescence-based quantification, a PacBio Sequel sequencer was utilized for sequencing. The bacterial 16 S rRNA gene V3–V4 region was amplified with primers 341 F and 806R, and sequenced on the Illumina NovaSeq 6000 platform (2 × 250 bp paired-end). Raw reads were demultiplexed, quality-filtered (Q ≥ 20), merged (minimum overlap 50 bp), and clustered into amplicon sequence variants (ASVs) with DADA2; chimeras were removed using the consensus method. Taxonomy was assigned against the SILVA 138 database with a confidence threshold of 80%.

Thereafter, the sequences were subjected to denoising through the QIIME2 dada2 analysis pipeline. After sequences with an abundance of less than five were filtered out, the final set of sequence variants, referred to as single sequence variants (SVs), along with their characteristic table, were obtained. This allowed for the analysis of the cecum content microflora in Leizhou black ducks.

### Statistical analysis

Statistical analysis was conducted with the pen as the experimental unit (*n* = 10 ducks per pen) for all growth-performance traits; for microbiome and meat-quality determinations, six birds were randomly sampled from each pen at slaughter, and all data were expressed as pen means to maintain unit consistency, ensure adequate replication, and avoid pseudo replication.

The immunoblotting images used were the clearest ones obtained from repeated analyses and were quantified by NIH ImageJ software (ImageJ 149, NIH, Bethesda, MD, USA). SPSS 26.0 was used for one-way ANOVA, followed by Tukey’s HSD post hoc test. Normality was tested with the Shapiro–Wilk test (W > 0.95 and *P* > 0.05). Levene’s test confirmed homogeneity of variances before ANOVA. Statistical significance was set at *p* < 0.05, indicating a significant difference, and *p* < 0.01 denoted an extremely significant difference. Results were expressed as mean ± SD.

To explore the relationships between the gut microbiota (classified at the phylum to genus level) and Leizhou black duck production performance, meat quality, flavor compounds, and intestinal health, Spearman’s rank correlation analysis was performed via the OmicStudio tools accessible at https://www.omicstudio.cn/tool. A p value less than 0.05 was considered indicative of a statistically significant difference, whereas a trend approaching significance was defined as 0.05 < *P* < 0.10.

## Results

### Enhanced growth performance in Leizhou black ducks administered Rhodotorula mucilaginosa ZTHY2

Animal feeding experiments were performed to evaluate the effects of ZTHY2 on the growth performance of Leizhou black ducks. Table 2 showed that during the initial 21 days, the 2 × 10^9^ CFU/kg RM group had significantly greater ADG and ADFI values than the control group (*P* < 0.05). Compared with the control group, the 2 × 10^8^ CFU/kg and 2 × 10^9^ CFU/kg RM groups also presented a significant increase in ADG and a decrease in FCR during days 21–42 (*P* < 0.01 and *P* < 0.05, respectively). Throughout the entire 42-day experiment, the ZTHY2 yeast and 2 × 10^9^ CFU/kg LA groups presented greater ADG than did the control group (*P* < 0.05), whereas the 2 × 10^8^ CFU/kg and 2 × 10^9^ CFU/kg RM groups presented a significant reduction in FCR (*P* < 0.05). No significant differences in ADF were found between the ZTHY2 yeast-treated groups and the control in either the 21-42-day period or the full 1-42-day period (*P* > 0.05).

**Table 2:** Effect of R. mucilaginosa ZTHY2 on the growth performance of Leizhou black ducks

Items	Control	RM1	RM2	RM3	LA
*1-21d*					
ADFI(g/d)	73.27 ± 1.66^b^	75.20 ± 0.16^ab^	74.35 ± 1.18^ab^	76.21 ± 0.69^a^	73.24 ± 1.11^b^
ADG(g/d)	35.12 ± 1.40^b^	36.80 ± 3.22^ab^	37.79 ± 3.12^ab^	39.35 ± 2.40^a^	36.36 ± 2.64^ab^
FCR	2.09 ± 0.08	2.06 ± 0.18	1.98 ± 0.17	1.94 ± 0.12	2.02 ± 0.15
*21-42d*					
ADFI(g/d)	200.17 ± 1.20	207.70 ± 5.90	208.40 ± 4.67	208.94 ± 4.20	204.67 ± 7.30
ADG(g/d)	31.43 ± 1.77^Bc^	35.39 ± 2.14^ABbc^	40.77 ± 1.02^Aab^	41.30 ± 1.93^Aa^	35.72 ± 2.19^ABabc^
FCR	6.48 ± 0.39^a^	5.97 ± 0.33^ab^	5.13 ± 0.13^b^	5.12 ± 0.25^b^	5.85 ± 0.41^ab^
*1-42d*					
ADFI(g/d)	136.72 ± 1.07	141.45 ± 2.95	141.37 ± 2.66	142.58 ± 2.28	138.96 ± 3.95
ADG(g/d)	33.27 ± 0.96^Bc^	36.10 ± 0.63^Bb^	39.28 ± 0.70^Aa^	40.33 ± 0.83^Aa^	36.04 ± 0.74^Bb^
FCR	4.13 ± 0.13^Aa^	3.92 ± 0.07^ABab^	3.60 ± 0.06^BCc^	3.54 ± 0.07^Cc^	3.86 ± 0.08^ABCb^

In peer data, different lowercase letters (abc) of shoulder label indicate significant difference (*P <* 0.05), different uppercase letters (ABC) of shoulder label indicate very significant difference (*P <* 0.01), and the same or no lowercase letters indicate no significant difference (*P >* 0.05)

### Influence of ZTHY2 on the slaughter performance of Leizhou black ducks

The slaughter performance of Leizhou black meat ducks was the primary focus of this study. Table 3 showed that adding 2 × 10^9^ CFU/kg ZTHY2 to the duck diet significantly improved Leizhou black duck slaughter performance (*P* < 0.05), with increasing in evisceration, semievisceration, breast, and leg muscle rates, but these improvements were not statistically significant (*P* > 0.05).

**Table 3:** Effect of R. mucilaginosa ZTHY2 on the slaughter performance of Leizhou black ducks (%)

Items	Control	RM1	RM2	RM3	LA
Slaughter rate (%)	85.22 ± 0.47^b^	85.86 ± 0.11 ^b^	87.51 ± 0.43 ^ab^	88.62 ± 0.57 ^a^	87.12 ± 1.43 ^ab^
Full bore rate (%)	72.80 ± 0.83	73.69 ± 0.40	73.78 ± 0.76	76.14 ± 2.42	73.73 ± 1.27
Half bore rate (%)	79.51 ± 0.74	80.48 ± 0.30	80.57 ± 1.21	83.45 ± 2.64	81.31 ± 1.43
Chest muscle percentage (%)	11.20 ± 0.38	11.30 ± 0.15	11.17 ± 0.61	12.52 ± 1.52	11.32 ± 0.29
Leg muscle percentage (%)	12.42 ± 0.32	12.75 ± 0.27	12.8 ± 0.27	13.02 ± 0.10	12.87 ± 0.28

In peer data, different lowercase letters (abc) of shoulder label indicate significant difference (*P <* 0.05), and the same or no lowercase letters indicate no significant difference (*P >* 0.05)

### The impact of ZTHY2 on the quality and characteristics of Leizhou black Duck meat

The incorporation of 2 × 10^9^ CFU/kg ZTHY2 into duck feed noticeably reduced meat brightness (L*) and yellowness (b*) in both the breast and leg muscles but significantly increased breast muscle redness (a*) (*P* < 0.05), as shown in Table 4. Moreover, 2 × 10^9^ CFU/kg ZTHY2 significantly increased the pH_24h_ value in Leizhou black duck breast muscle (*P* < 0.05), significantly increasing the pH_24h_ value in duck leg muscle (*P* < 0.01). Additionally, compared with the control group, the 2 × 10^9^ CFU/kg RM group presented a significant reduction in the water loss rate in both the breast and leg muscles (*P* < 0.05).

**Table 4:** Effect of R. mucilaginosa ZTHY2 on the meat quality of Leizhou black ducks

Items		Control		RM1			RM2		RM3		LA	
Chest muscle	L*	48.11 ± 2.96^a^		47.73 ± 0.66^a^			43.66 ± 5.13^ab^		40.40 ± 3.98^b^		44.80 ± 3.32^ab^	
	a*	8.96 ± 2.55		9.02 ± 1.28			10.79 ± 0.82		10.76 ± 1.48		10.03 ± 1.10	
	b*	14.59 ± 1.25^Aa^		11.81 ± 1.90^ABb^			11.70 ± 0.36^ABb^		10.46 ± 0.76^Bb^		13.21 ± 2.11^ABab^	
	pH_45min_	5.95 ± 0.62		6.07 ± 0.32			6.40 ± 0.06		6.41 ± 0.15		6.32 ± 0.20	
	pH_24h_	5.89 ± 0.04^b^		5.90 ± 0.08^b^			5.95 ± 0.02^b^		6.31 ± 0.30^a^		5.90 ± 0.03^b^	
	Water loss rate (%)	28.55 ± 2.60^a^		26.99 ± 1.32^a^			22.54 ± 3.26^ab^		19.48 ± 5.65^b^		24.64 ± 4.10^ab^	
	Cooking loss (%)	35.67 ± 2.36		35.40 ± 4.12			34.83 ± 1.88		32.57 ± 1.15		34.67 ± 2.11	
	Shear force(N)	25.09 ± 1.66		24.91 ± 2.38			21.74 ± 2.14		21.21 ± 1.22		24.91 ± 2.67	
Leg muscle	L*	49.02 ± 0.49^a^		48.19 ± 1.20^ab^			45.28 ± 0.33^ab^		44.76 ± 1.05^b^		45.21 ± 4.46^ab^	
	a*	6.98 ± 1.00^b^		7.59 ± 0.59^ab^			8.93 ± 0.72^ab^		8.98 ± 1.74^b^		8.70 ± 0.44^ab^	
	b*		12.80 ± 1.15^a^		11.00 ± 0.72^ab^	11.01 ± 1.23^ab^		10.12 ± 0.77^b^		10.12 ± 0.78^ab^		
	pH_45min_		6.28 ± 0.17		6.08 ± 0.13	6.39 ± 0.10		6.44 ± 0.05		6.39 ± 0.42		
	pH_24h_		5.91 ± 0.13^Bb^		5.93 ± 0.06^Bb^	5.95 ± 0.03^Bb^		6.25 ± 0.08^Aa^		5.92 ± 0.04^Bb^		
	Water loss rate (%)		23.73 ± 5.24^a^		21.97 ± 0.28^a^	19.2 ± 1.99^ab^		15.86 ± 1.83^b^		22.27 ± 2.31^a^		
	Cooking loss (%)		29.89 ± 1.68		29.53 ± 1.26	28.70 ± 2.40		24.95 ± 4.36		27.14 ± 2.77		
	Shear force(N)		30.54 ± 2.29		27.12 ± 3.05	26.60 ± 2.65		26.81 ± 0.91		27.40 ± 2.09		

In peer data, different lowercase letters (abc) of shoulder label indicate significant difference (*P* < 0.05), different uppercase letters (ABC) of shoulder label indicate very significant difference (P < 0.01), and the same or no lowercase letters indicate no significant difference (*P* > 0.05)

### Influence of ZTHY2 on the nutritional composition of conventional nutrients in Leizhou black ducks

The general nutrient composition of the muscle of Leizhou black duck muscle formed the basis for meat quality. Table 5 showed the effects of supplementing the conventional feed of Leizhou black ducks with ZTHY2. Compared with the control group, supplementation with 2 × 10^9^ CFU/kg RM and 2 × 10^9^ CFU/kg LA significantly elevated the crude protein content in both the breast and leg muscles (*P* < 0.05). Furthermore, the inclusion of 2 × 10^7^ CFU/kg and 2 × 10^9^ CFU/kg RM as well as 2 × 10^9^ CFU/kg LA in the duck feed resulted in a notable reduction in the crude fat content in duck breast muscle (*P* < 0.05). However, ZTHY2 had no significant effect on the water or ash content of Leizhou black duck meat (*P* > 0.05).

**Table 5:** Effect of R. mucilaginosa ZTHY2 on the muscle general nutrient content of Leizhou black ducks

Items		Control	RM1	RM2	RM3	LA
Chest muscle	moisture(%)	76.63 ± 0.39	75.44 ± 0.31	75.09 ± 1.07	74.67 ± 0.78	75.32 ± 2.02
	crude protein(%)	20.63 ± 0.25^b^	21.31 ± 0.5^ab^	21.37 ± 0.34^ab^	22.42 ± 0.26^a^	21.91 ± 0.44^a^
	crude fat(%)	2.07 ± 0.02^a^	1.40 ± 0.21^b^	1.84 ± 0.09^a^	1.36 ± 0.05^b^	1.41 ± 0.06^b^
	crude ash(%)	1.35 ± 0.02	1.36 ± 0.12	1.44 ± 0.02	1.46 ± 0.05	1.52 ± 0.01
Leg muscle	moisture(%)	76.02 ± 0.22	75.20 ± 0.68	75.07 ± 0.59	75.10 ± 0.51	75.48 ± 0.54
	crude protein(%)	20.57 ± 0.33^b^	21.28 ± 0.45^ab^	21.52 ± 0.21^ab^	21.80 ± 0.36^a^	21.45 ± 0.13^ab^
	crude fat(%)	1.78 ± 0.03	1.76 ± 0.08	1.77 ± 0.04	1.76 ± 0.04	1.73 ± 0.04
	crude ash(%)	1.35 ± 0.01	1.42 ± 0.09	1.37 ± 0.04	1.32 ± 0.04	1.40 ± 0.06

In peer data, different lowercase letters (abc) of shoulder label indicate significant difference (*P* < 0.05), different uppercase letters (ABC) of shoulder label indicate very significant difference (P < 0.01), and the same or no lowercase letters indicate no significant difference (*P* > 0.05)

### The influence of ZTHY2 on alterations in the amino acid and fatty acid profiles of Leizhou black duck muscle tissue

Changes in amino acid and fatty acid composition affected the muscle flavor of Leizhou black ducks. Table 6 showed the impact of ZTHY2 feeding on the amino acid and fatty acid profiles of muscle tissue from Leizhou black ducks. Notably, the serine and tyrosine concentrations in the leg muscles of these ducks significantly increased (*P* < 0.05) when the feed was supplemented with 2 × 10^9^ CFU/kg ZTHY2 compared with those of the control group. Conversely, a notable decrease (*P* < 0.05) in lysine content was detected when the same group was given 2 × 10^9^ CFU/kg LA. Additionally, the levels of total free amino acids (TFAAs), essential amino acids (EAAs), nonessential amino acids (NEAAs), and dispensable amino acids (DAAs) were generally greater across all groups that received different concentrations of ZTHY2, but these differences were not statistically significant (*P* > 0.05). Table 7 shows the effects of ZTHY2 on the fatty acid composition of Leizhou black duck muscle. Compared with the control group, the 2 × 10^7^ CFU/kg, 2 × 10^8^ CFU/kg, and 2 × 10^9^ CFU/kg RM groups, showed significant decreases in the nervonic acid content (*P* < 0.05). Additionally, the LA group at a 2 × 10^9^ CFU/kg dosage presented significant reductions in the proportions of myristic acid, nervonic acid, and linoleic acid (*P* < 0.05). The inclusion of ZTHY2 in the diet resulted in a decrease in saturated fatty acids (SFAs) and polyunsaturated fatty acids (PUFAs) and an increase in monounsaturated fatty acids (MUFAs) in the muscle tissue, but these changes were not statistically significant (*P* > 0.05).

**Table 6:** Effect of R. mucilaginosa ZTHY2 on the muscle amino acid contents of Leizhou black ducks (%)

Items		Control	RM1	RM2	RM3	LA
EAA	Thr	0.65 ± 0.06^b^	0.73 ± 0.11^ab^	0.72 ± 0.11^ab^	0.79 ± 0.04^a^	0.72 ± 0.11^ab^
	Val	0.75 ± 0.06	0.75 ± 0.12	0.78 ± 0.11	0.84 ± 0.06	0.75 ± 0.16
	Met	0.37 ± 0.03^a^	0.37 ± 0.07^a^	0.35 ± 0.12^ab^	0.38 ± 0.07^a^	0.27 ± 0.08^b^
	Ile	0.66 ± 0.07	0.68 ± 0.13	0.68 ± 0.13	0.72 ± 0.09	0.66 ± 0.16
	Leu	1.20 ± 0.11	1.25 ± 0.21	1.27 ± 0.21	1.40 ± 0.10	1.26 ± 0.25
	Lys	1.47 ± 0.15	1.51 ± 0.22	1.62 ± 0.29	1.61 ± 0.10	1.59 ± 0.22
	Phe	0.59 ± 0.05	0.62 ± 0.11	0.66 ± 0.10	0.69 ± 0.04	0.65 ± 0.09
NEAA	Gly	0.67 ± 0.11	0.69 ± 0.10	0.71 ± 0.08	0.72 ± 0.04	0.68 ± 0.10
	Ala	0.97 ± 0.11	1.00 ± 0.15	1.02 ± 0.18	1.12 ± 0.06	1.04 ± 0.18
	Glu	2.05 ± 0.56	2.19 ± 0.55	2.05 ± 0.56	2.44 ± 0.45	1.99 ± 0.62
	Asp	1.24 ± 0.30	1.34 ± 0.31	1.33 ± 0.33	1.52 ± 0.21	1.33 ± 0.30
	Tyr	0.53 ± 0.06^b^	0.56 ± 0.10^ab^	0.58 ± 0.09^ab^	0.66 ± 0.05^a^	0.59 ± 0.10^ab^
	Ser	0.63 ± 0.07	0.68 ± 0.11	0.68 ± 0.09	0.73 ± 0.03	0.69 ± 0.07
	His	1.56 ± 0.56	1.49 ± 0.41	2.03 ± 1.01	1.43 ± 0.44	1.83 ± 1.03
	Pro	0.43 ± 0.08	0.41 ± 0.10	0.44 ± 0.07	0.47 ± 0.06	0.46 ± 0.10
	Arg	1.01 ± 0.21	0.97 ± 0.16	1.06 ± 0.20	1.09 ± 0.10	0.98 ± 0.18
Nutritional evaluation	TFAA	14.77 ± 1.13	15.25 ± 2.00	15.97 ± 2.06	16.62 ± 0.98	15.47 ± 2.01
	EAA	5.68 ± 0.40	5.90 ± 0.94	6.09 ± 0.85	6.43 ± 0.38	5.89 ± 0.96
	NEAA	9.09 ± 0.77	9.34 ± 1.05	9.9 ± 1.28	10.18 ± 0.61	9.58 ± 1.12
	DAA	6.04 ± 1.00	6.40 ± 1.27	6.35 ± 1.25	7.15 ± 0.78	6.28 ± 1.37
	EAA/TFAA	38.46 ± 0.66	38.55 ± 1.14	38.09 ± 1.60	38.68 ± 0.56	37.99 ± 2.04
	NEAA/TFAA	61.53 ± 0.71	61.37 ± 1.17	62.01 ± 1.64	61.28 ± 0.55	62.01 ± 2.02
	EAA/NEAA	62.53 ± 1.76	62.86 ± 3.06	61.52 ± 4.15	63.14 ± 1.48	61.4 ± 5.19
	DAA/TFAA	40.85 ± 5.36	41.70 ± 3.43	39.65 ± 4.56	42.95 ± 2.66	40.27 ± 4.75

In peer data, different lowercase letters (abc) of shoulder label indicate significant difference (*P <* 0.05), and the same or no lowercase letters indicate no significant difference (*P >* 0.05). DAA include: Glu, Ala, Asp, Tyr, Phe, Tyr

**Table 7:** Effect of R. mucilaginosa ZTHY2 on the muscle fatty acid content of Leizhou black ducks

Items		Control		RM1	RM2			RM3	LA
Saturated fatty acid(SFA)									
Myristic acid C14:0	0.49 ± 0.01^a^		0.45 ± 0.03^ab^			0.48 ± 0.01^a^	0.49 ± 0.01^a^		0.43 ± 0.01^b^
Palmitic acid C16:0	21.51 ± 0.22		22.82 ± 0.72			23.05 ± 0.36	21.75 ± 0.17		22.69 ± 1.00
Stearic acid C18:0	10.67 ± 1.66		8.98 ± 0.42			8.26 ± 0.15	8.38 ± 1.27		8.69 ± 1.16
SFA	32.67 ± 2.68		32.25 ± 1.37			31.79 ± 0.76	30.61 ± 2.47		31.81 ± 1.18
Monounsaturated fatty acids(MUFA)									
Palmitoleic acid C16:1n-7	1.71 ± 0.13		1.80 ± 0.11			2.23 ± 0.04	2.23 ± 0.19		2.22 ± 0.32
Oleic acid C18:1n9	36.19 ± 2.22		38.29 ± 1.22			40.04 ± 0.13	40.51 ± 2.97		40.59 ± 1.81
Cis-11-eicosenoic acid C20:1	0.23 ± 0.02		0.23 ± 0.01			0.27 ± 0.04	0.28 ± 0.03		0.25 ± 0.02
Cis-11, 14-eicosadienoic acid C20:2	0.37 ± 0.04		0.35 ± 0.02			0.38 ± 0.04	0.49 ± 0.05		0.42 ± 0.08
Cis-8,11, 14-eicosatrienoic acid C20:3n6	0.33 ± 0.08		0.31 ± 0.03			0.33 ± 0.06	0.33 ± 0.08		0.32 ± 0.08
Nervonic acid C24:1	1.14 ± 0.31^a^		0.41 ± 0.07^b^			0.40 ± 0.09 ^b^	0.41 ± 0.13 ^b^		0.31 ± 0.09 ^b^
MUFA	39.97 ± 3.36		41.38 ± 1.94			43.65 ± 0.60	44.26 ± 4.96		44.10 ± 3.27
Polyunsaturated fatty acids(PUFA)									
Linoleic acid C18:2n6	22.30 ± 0.97^ab^		22.78 ± 0.55^a^			21.48 ± 0.61^ab^	21.31 ± 0.46^ab^		20.30 ± 0.67^b^
Alpha-linolenic acid C18:3n3	0.88 ± 0.06^ab^		0.92 ± 0.03^a^			0.92 ± 0.05^a^	0.94 ± 0.01^a^		0.79 ± 0.03^b^
Arachidonic acid C20:4n6	4.18 ± 1.41		2.67 ± 0.48			2.14 ± 0.44	2.87 ± 0.98		2.99 ± 0.87
PUFA	27.37 ± 0.68		26.37 ± 1.58			24.55 ± 0.58	25.13 ± 2.49		24.08 ± 2.47

In peer data, different lowercase letters (abc) of shoulder label indicate significant difference (*P* < 0.05), and the same or no lowercase letters indicate no significant difference (*P* > 0.05)

### Influence of ZTHY2 on the histological features and architectural integrity of the intestinal tract in Leizhou black ducks

**Fig. 1:**
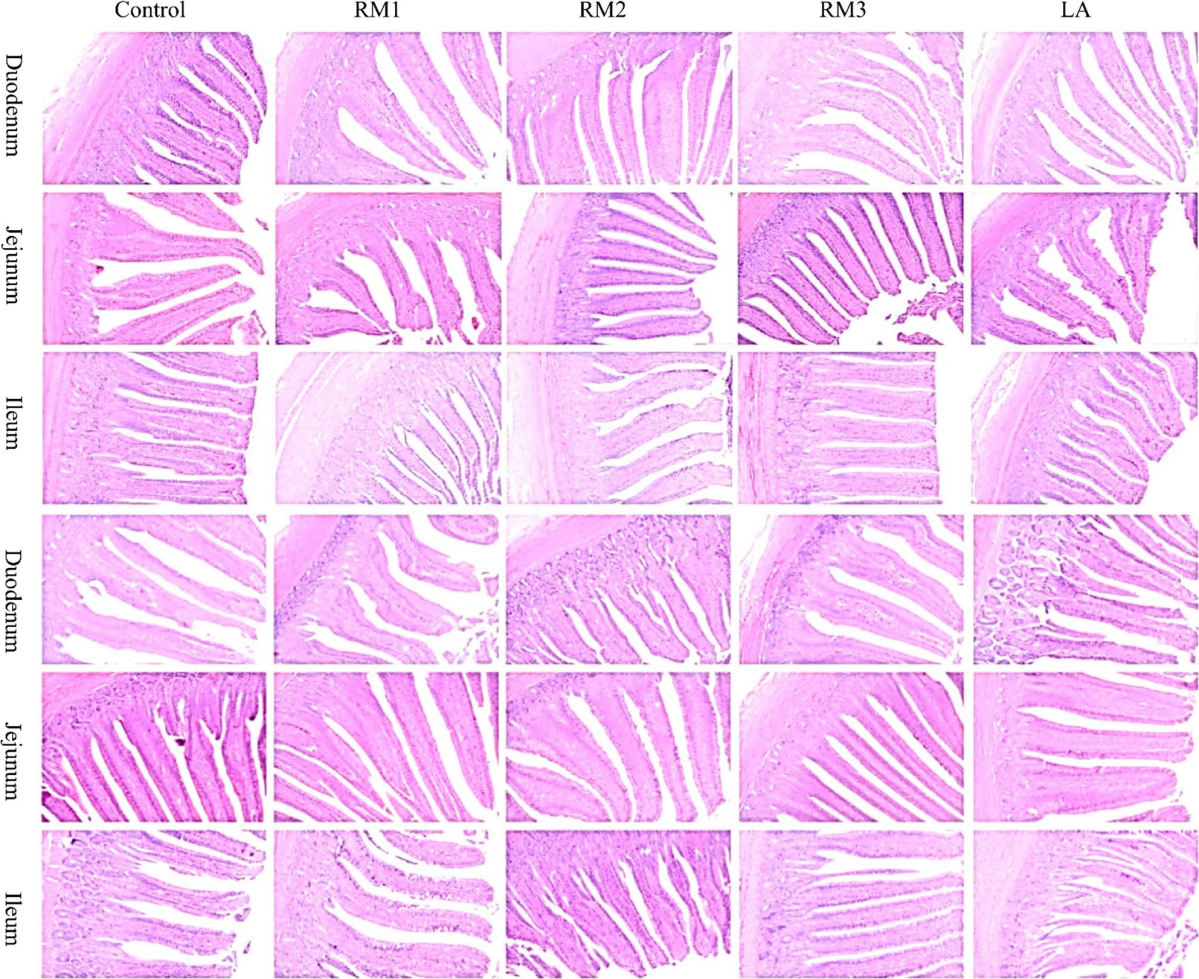
Morphology of the duodenum, jejunum, and ileum of 21-d-old and 42-d-old Leizhou black ducks. ZTHY2 preserved tall villi and shallow crypts, expanding absorptive surface along the small intestine. Control (Control group), RM1 (2 × 10^7^ CFU/kg RM group), RM2 (2 × 10^8^ CFU/kg RM group), RM3 (2 × 10^9^ CFU/kg RM group), LA (2 × 10^9^ CFU/kg LA group). RM, Rhodotorula mucilaginosa; LA, Lactobacillus acidophilus

In this study, we assessed the impact of ZTHY2 on the intestinal tract morphology of Leizhou black ducks, as shown in Fig. 1. Table 8 revealed that the incorporation of this yeast into the duck diet significantly increased the villus length and the villus-to-crypt ratio in the duodenum, jejunum, and ileum of 21- and 42-day-old ducks relative to those in the control group. Additionally, crypt depth in the jejunum was significantly reduced at both ages. The greatest effect was achieved at a yeast dosage of 2 × 10^9^ CFU/kg (*P* < 0.01). Microscopic examination revealed well-preserved, orderly villus structures with distinct crypts in all the treated groups, with no lesions. Yeast administration promoted tighter villus packing, enhancing the growth and proliferation of intestinal mucosal cells and maintaining a clear, intact mucosal architecture.

**Table 8:** Effect of R. mucilaginosa ZTHY2 on the intestinal morphology of Leizhou black ducks

Items	Control	RM1	RM2	RM3	LA
21d duodenum					
VH/µm	589.24 ± 37.37^Cc^	788.79 ± 42.73^Bb^	844.45 ± 25.62^ABb^	980.53 ± 36.37^Aa^	819.02 ± 32.17^Bb^
CD/µm	255.45 ± 17.70	259.05 ± 11.24	254.44 ± 17.32	241.94 ± 25.30	259.48 ± 16.22
V/C	2.37 ± 0.24^Bc^	3.07 ± 0.21^Bcb^	3.39 ± 0.24^ABb^	4.20 ± 0.31^Aa^	3.21 ± 0.22^ABb^
42d duodenum					
VH/µm	892.73 ± 75.19^b^	788.79 ± 42.73^ab^	893.43 ± 46.96^ab^	985.62 ± 23.82^a^	979.91 ± 35.04^a^
CD/µm	325.6 ± 28.87^Aa^	232.39 ± 15.96^Bb^	256.58 ± 14.72^ABb^	225.67 ± 10.21^Bb^	256.42 ± 15.86^ABb^
V/C	2.91 ± 0.40^Bc^	3.45 ± 0.23^ABbc^	3.59 ± 0.38^ABabc^	4.39 ± 0.13^Aa^	3.87 ± 0.19^ABab^
21d jejunum					
VH/µm	577.82 ± 16.77^Bc^	610.46 ± 63.51^Bbc^	624.46 ± 24.31^Bbc^	876.58 ± 37.39^Aa^	699.26 ± 28.76^Bb^
CD/µm	243.49 ± 23.38^Aa^	220.31 ± 12.94^ABa^	214.48 ± 12.18^ABa^	159.29 ± 10.02^Bb^	208.6 ± 25.23^ABab^
V/C	2.50 ± 0.28^Bc^	2.77 ± 0.22^Bbc^	2.95 ± 0.17^Bbc^	5.56 ± 0.26^Aa^	3.53 ± 0.32^Bb^
42d jejunum					
vVH/µm	991.23 ± 18.9^Bb^	1149.64 ± 22.65^a^	1211.15 ± 43.03^a^	1227.93 ± 61.54^a^	1202.87 ± 37.3^a^
CD/µm	238.37 ± 13.59^Aa^	224.82 ± 12.34^ABab^	209.89 ± 6.10^ABab^	144.58 ± 9.59^Cc^	194.19 ± 6.19^Bbc^
V/C	4.23 ± 0.24^Cc^	5.21 ± 0.36^BCbc^	5.81 ± 0.31^Bb^	8.61 ± 0.49^Aa^	6.21 ± 0.22^Bb^
21d ileum					
VH/µm	580.30 ± 16.99^Cc^	683.15 ± 12.29^ABb^	699.55 ± 23.14^Ab^	757.68 ± 29.16^Aa^	612.85 ± 10.96^BCc^
CD/µm	256.83 ± 14.97	251.42 ± 14.43	264.69 ± 15.44	257.98 ± 8.87	230.5 ± 7.20
V/C	2.31 ± 0.18^b^	2.76 ± 0.17^ab^	2.7 ± 0.22^ab^	2.97 ± 0.21^a^	2.67 ± 0.082^ab^
42d ileum					
VH/µm	595.10 ± 11.76^Cc^	702.17 ± 13.71^ABb^	726.9 ± 40.63^ABab^	769.53 ± 11.44^Aa^	675.39 ± 17.53^BCb^
CD/µm	280.92 ± 9.09	288.78 ± 9.27	275.66 ± 21.83	265.49 ± 16.58	275.78 ± 6.34
V/C	2.09 ± 0.08^Bc^	2.44 ± 0.09^ABbc^	2.67 ± 0.10^ABab^	2.97 ± 0.25^Aa^	2.45 ± 0.06^ABbc^

In peer data, different lowercase letters (abc) of shoulder label indicate significant difference (*P <* 0.05), different uppercase letters (ABC) of shoulder label indicate very significant difference (*P <* 0.01), and the same or no lowercase letters indicate no significant difference (*P >* 0.05). VH: intestinal villus height; CD: crypt depth; V/C: the fleece-to-crypt ratio

### Influence of Rhodotorula mucilaginosa ZTHY2 on the intestinal microbiota composition of Leizhou black ducks

Sequence alignment and OTU validation analysis.

**Fig. 2:**
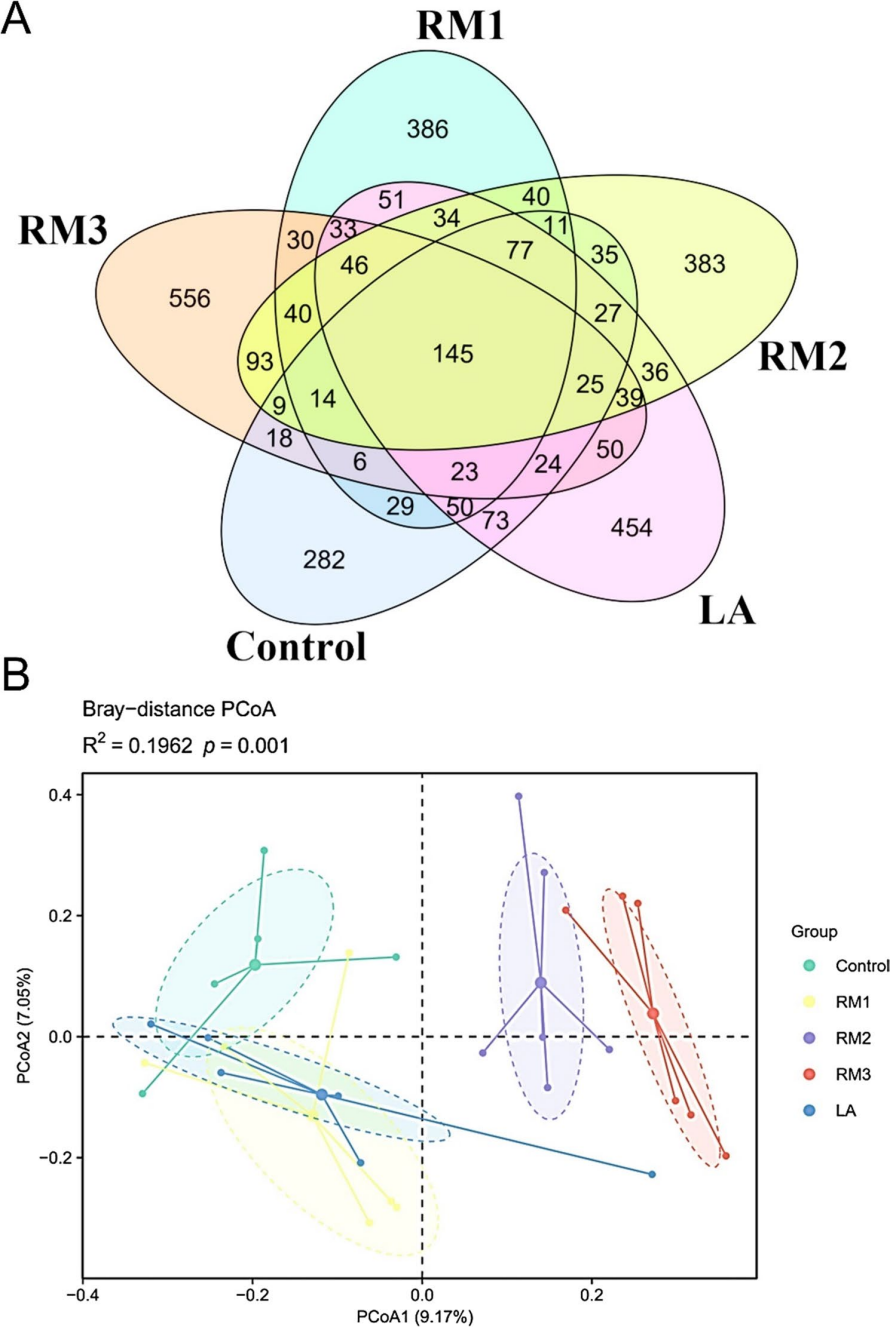
Validity analysis of the cecal microbiota in Leizhou black ducks. Core OTUs expanded and beta-diversity tightened under ZTHY2, denoting stable, beneficial cecal communities. **A** Venn diagram; **B** Beta diversity analysis. Control (Control group), RM1 (2 × 10^7^ CFU/kg RM group), RM2 (2 × 10^8^ CFU/kg RM group), RM3 (2 × 10^9^ CFU/kg RM group), LA (2 × 10^9^ CFU/kg LA group). RM, Rhodotorula mucilaginosa; LA, Lactobacillus acidophilus

In the present study, we utilized high-throughput sequencing to examine and contrast the cecum microbiota of Leizhou black ducks from the various treatments. As illustrated in Fig. 2A, the control group, RM1 group, RM2 group, RM3 group, and LA group contained 145, 282, 386, 383, and 454 unique ASVs, respectively. These results indicated that the composition of the cecal microbiota in Leizhou black ducks could be modified through the inclusion of Saccharomyces cerevisiae in their diet.

The Chao1 index was a measure of species richness in a sample, and higher values indicated greater diversity within a community. Figure s1A showedthat the difference in the Chao1 index was not statistically significant across groups, implying similar species richness. However, both the RM and LA groups presented higher Chao1 indices than did the control group. In contrast, the Shannon index, which quantifies species diversity, did not significantly differ among the groups, suggesting consistent diversity levels (Figure s1D). Compared with the control group, the RM group presented greater species richness and evenness (Figure s1A, B, C and D).

Principal coordinate analysis (PCoA) with the Bray‒Curtis dissimilarity metric was used to reveal the β diversity of the bacterial flora, as shown in Fig. 2B. Notable differences were found in bacterial composition among the groups, with an R^2^ of 0.2163 and a P value of 0.002. The lack of overlap between the control, RM1, and LA groups and the RM2 and RM3 groups highlights significant dissimilarities in bacterial community structure among these groups. Moreover, the distinct spatial separation of the RM3 group from the other clusters suggests substantial heterogeneity in the composition of the intestinal microbiota within the RM3 group.

Examination of floral structure and biodiversity across various taxonomic levels.

Through 16 S amplicon sequencing, we identified 3119 operational taxonomic units (OTUs) across 14 phyla in the intestinal bacteria of Leizhou black ducks (Figures s2 and s3). The ten most abundant taxa were Bacteroidota, Desulfobacterota, Firmicutes, Proteobacteria, Verrucomicrobiota, Fusobacteriota, Elusimicrobiota, Deferribacteres, Spirochaetota, and Cyanobacteria, indicating a prevalence of Bacteroidetes. Additionally, Proteobacteria were significantly less abundant in the RM3 group (*P* < 0.05) than in the control group, whereas Actinobacteria were significantly more abundant in the RM1 group (*P* < 0.01).

After the microbial floral structure at the class level was analyzed, the ten most abundant classes in terms of species composition were chosen for visualization. Figures s2 and s4 showed these bacteria to be Bacteroidia, Clostridia, Desulfovibrionia, Negativicutes, Bacilli, Gammaproteobacteria, Alphaproteobacteria, Fusobacteria, Deferribacteres, and Vampirovibrionia. Additionally, Figure s4 revealed a significant decrease in the relative abundances of α-proteobacteria and γ-proteobacteria in the RM3 group compared with those in the control group (*P* < 0.05).

Analysis of the bacterial floral structure at the order, family, and genus levels (Figures s5, s6, s7) revealed that ZTHY2 feed supplementation significantly reduced the relative abundances of Clostridia_UCG-014, Rikenellaceae, Eubacterium coprostanoligenes_group, and their associated bacteria (*P* < 0.05) and concurrently increased the relative abundance of Oscillospiraceae (*P* < 0.05).

Linear discriminant analysis on intestinal flora.

Linear discriminant analysis, also known as LEfSe multilevel species difference analysis, was an analytical tool used to discover and interpret biological features (taxa, pathways, genes) of high-latitude datasets. Linear discriminant analysis (LDA), widely used in LEfSe for distinguishing species at multiple levels, was a powerful tool for identifying biological traits, including taxonomic groups, metabolic pathways, and gene expression profiles, within high-latitude datasets. It enabled comparisons between and within groups, highlighting significant biological differences. For example, it helped identify gut microbiota biomarkers among the five distinct groups in this study. Taxonomic classifications with LDA scores above 2 were graphically represented in bar charts, with LDA values used as a measure of the impact of each taxon on the observed gut microbiota discrepancies between groups; a higher score suggested a more significant contribution to intergroup differences in the gut microbiota composition.

**Fig. 3:**
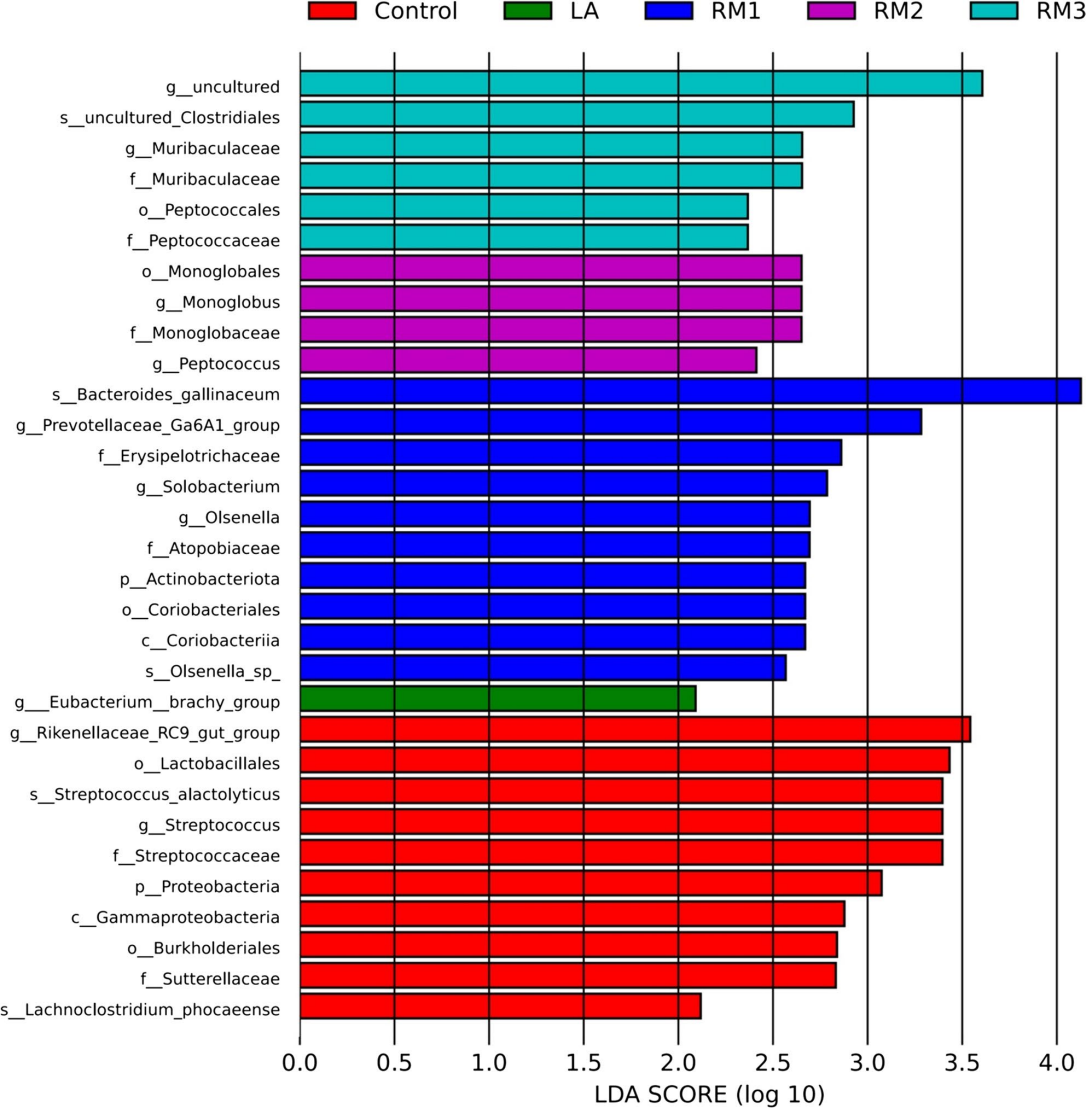
LEfSe multilevel discriminant analyses of species differences in Leizhou black ducks. ZTHY2 enriched Spirillaceae (B12 synthesis) and depleted LPS-rich Fusobacteriaceae, linking microbiota shift to reduced inflammation. Control (Control group), RM1 (2 × 10^7^ CFU/kg RM group), RM2 (2 × 10^8^ CFU/kg RM group), RM3 (2 × 10^9^ CFU/kg RM group), LA (2 × 10^9^ CFU/kg LA group). RM, Rhodotorula mucilaginosa; LA, Lactobacillus acidophilus

Figure 3 showed significant variations in microbial composition across treatment groups, highlighting notable differences in two phyla, two classes, five orders, five families, twelve genera, and five species. These taxonomic differences could function as diagnostic biomarkers for distinguishing among groups. In the control group, analysis revealed one phylum, one class, two orders, two families, two genera, and two species, including Rikenellaceae_RC9_gut_group, Lactobacillus, Streptococcus_alactolyticus, and Lachnoclostridium_phoca eense, among others. The RM1 group included one phylum, one class, one order, two families, four genera, and two species, with Prevotellaceae_Ga6A1_group, olobacterium, Olsenella, Bacteroides_gallinaceum, and Olsenella representing microorganisms at the genera and species levels, respectively. The RM2 group had one order, one family, and two genera, with Monoglobus and Peptococcus at the genus level. The RM3 group revealed one order, two families, and one genus, with Muribaculaceae as the identified genus. Finally, the LA group contained one genus, specifically Eubacterium_brachy_group.

LEfSe analysis indicated that the ZTHY2-supplemented group was enriched in Spirillaceae and depleted of Fusobacteriaceae compared with the control. Although cause-and-effect relationships cannot be ascertained from the present design, it is plausible that the decline in Fusobacteriaceae lowers the luminal concentration of lipopolysaccharide, thereby attenuating TLR4-mediated inflammatory signalling and sparing amino acids that would otherwise be oxidised during the acute-phase response. Concurrently, the expansion of Spirillaceae, many of which possess the cob operon, may enhance de-novo synthesis of vitamin B12; this cofactor is indispensable for methyl-group transfer in the methionine–homocysteine cycle and could thus increase the availability of methionine for muscle protein accretion. Taken together, these taxonomic shifts suggest a microbiota-mediated, anti-inflammatory and anabolic milieu that contributes to the improved breast-muscle yield and water-holding capacity observed in ZTHY2-fed ducks.

### Analysis of the correlations between intestinal flora and intestinal health, meat production performance, meat quality, and flavor substances in Leizhou black ducks with Rhodotorula mucilaginosa ZTHY2 feed supplementation

**Fig. 4:**
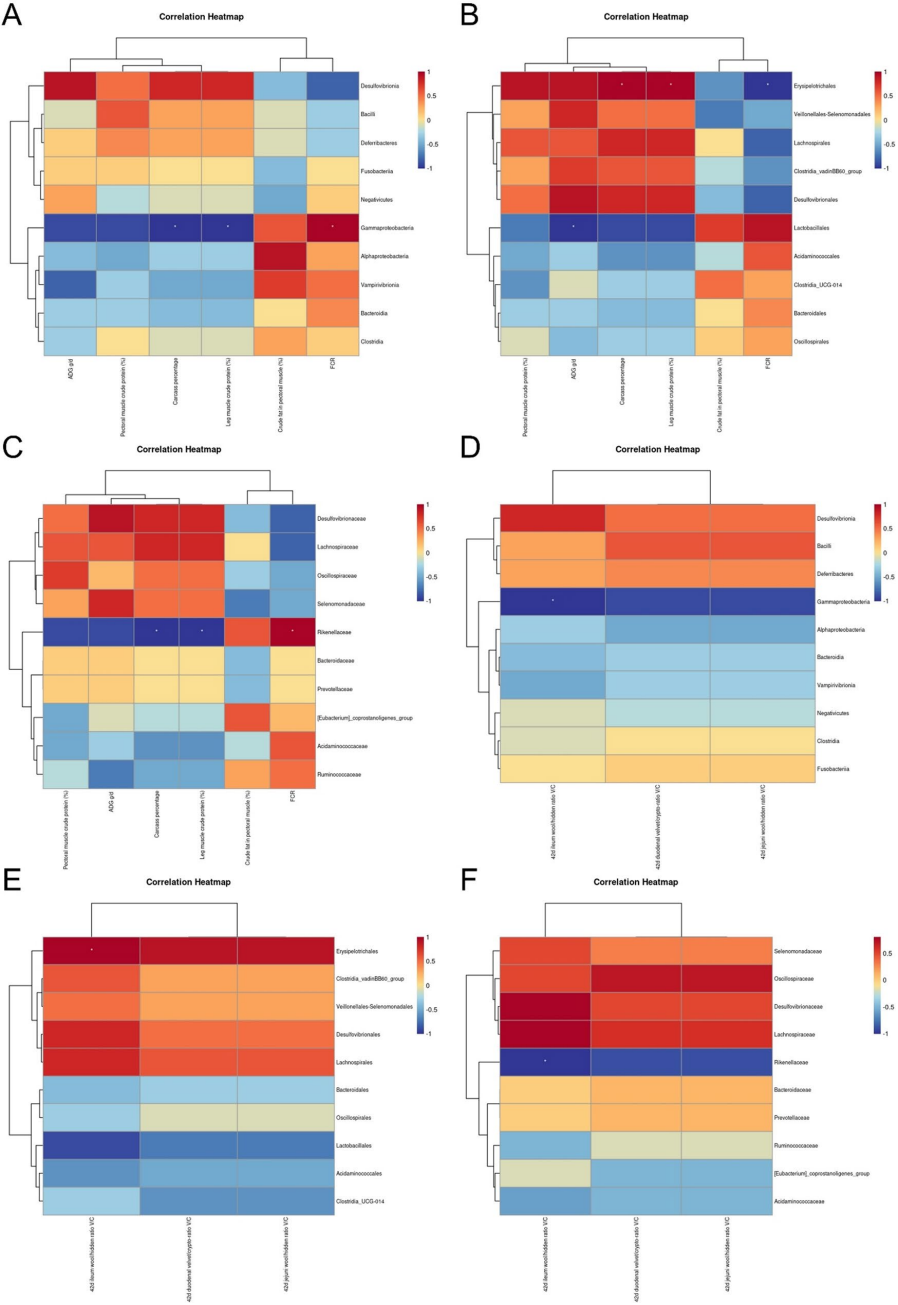
Spearman’s correlation analysis between the gut microbiota and meat performance and the intestinal health of Leizhou black ducks at the class (**A, D**), order (**B, E**) and family (**C, F**) levels. Spirillaceae positively correlated with villus height and breast-muscle yield, Fusobacteriaceae with poor FDR and higher endotoxin. Meat performance: class (**A**), order (**B**) and family (**C**); intestinal health: class (**D**), order (**E**) and family (**F**)

Spearman’s correlation analysis revealed significant relationships of Rhodotorula mucilaginosa ZTHY2 feed supplementation with meat production performance. At the class level, Gammaproteobacteria was positively correlated with the feed conversion ratio (FCR) (*P* < 0.05) and negatively correlated with the average daily gain (ADG) (g/d) and crude protein content in leg muscle (*P* < 0.05) (Fig. 4A). At the order level, Erysipelotrichales was positively associated with carcass percentage and leg muscle crude protein content but negatively correlated with FCR (*P* < 0.05). Lactobacillales was significantly negatively correlated with ADG (g/d) (*P* < 0.01) (Fig. 4B). At the family level, Rikenellaceae was positively correlated with FCR (*P* < 0.05) and negatively correlated with carcass percentage and leg muscle crude protein content (*P* < 0.05) (Fig. 4C).

Spearman’s rank correlation analysis revealed a significant negative relationship (*P* < 0.05) between the 42-day ileum wool/hidden ratio V/C measurements and the relative abundance of Gammaproteobacteria at the class level (Fig. 4D). Conversely, at the order level, a significant positive correlation (*P* < 0.05) was detected between the abundance of Erysipelotrichales and the 42-day ileum wool/hidden ratio V/C (Fig. 4E). Additionally, at the family level, a significant negative correlation (*P* < 0.05) was observed between Rikenellaceae and the 42-day ileum wool/hidden ratio V/C (Fig. 4F).

**Fig. 5:**
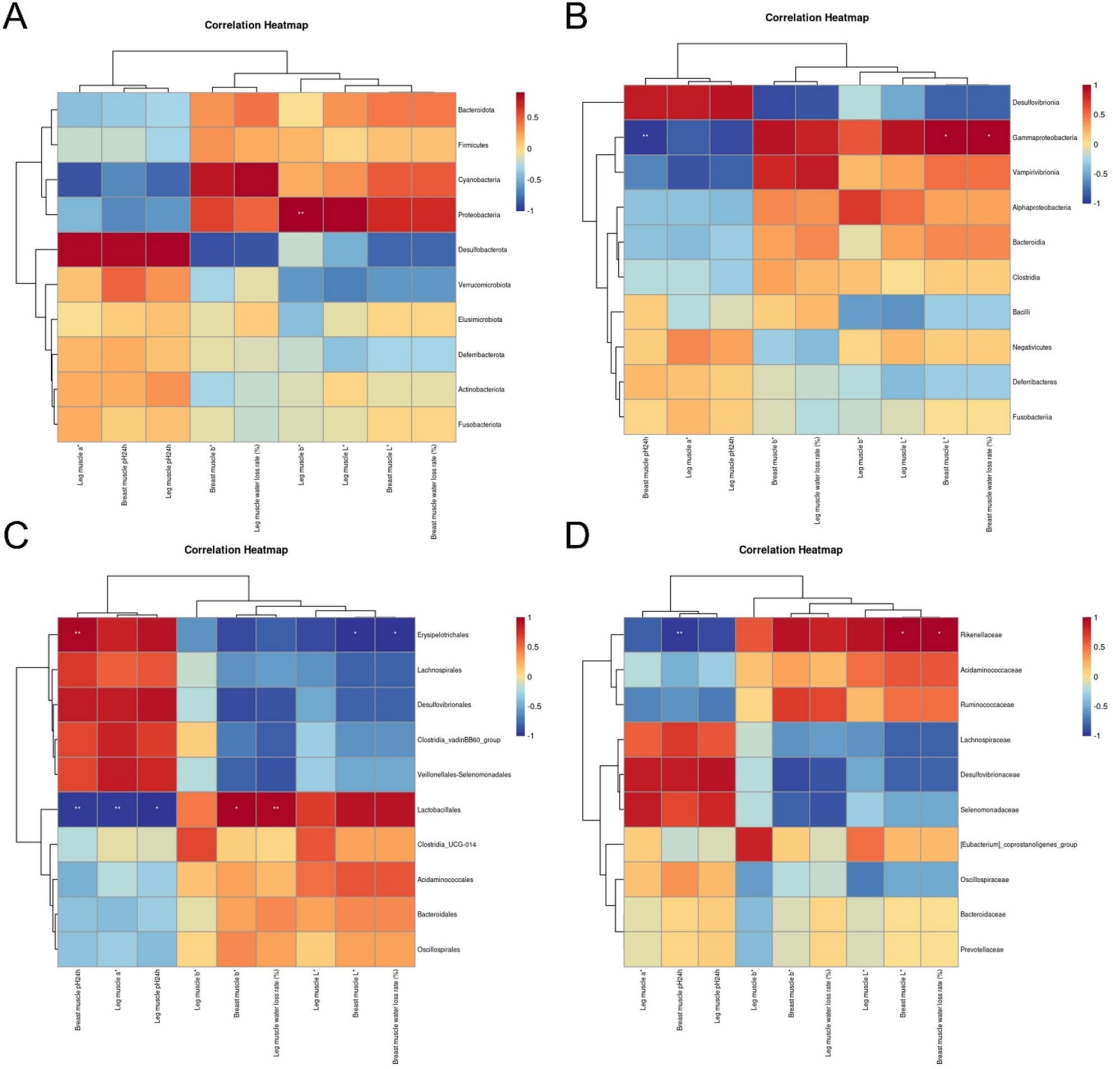
Spearman’s correlation analysis between the gut microbiota and meat quality of Leizhou black duck at the phylum (**A**), class (**B**), order (**C**) and family (**D**) levels. Higher Spirillaceae associated with lighter color and lower drip loss; Fusobacteriaceae linked to elevated b* and rapid pH decline

In terms of meat quality, at the phylum level, Spearman’s correlation analysis revealed a significant positive relationship between the abundance of Proteobacteria and the recorded values of leg muscle b* (*P* < 0.01), as shown in Fig. 5A. At the class level, Gammaproteobacteria was positively correlated with breast muscle L* and the water loss rate (%) but negatively correlated with breast muscle pH24h (*P* < 0.05 and *P* < 0.01, respectively), as depicted in Fig. 5B. At the class level, Erysipelotrichales was found to be positively associated with the breast muscle pH24h (*P* < 0.01) but negatively correlated with the breast muscle L* and water loss rate (%) (*P* < 0.05), as shown in Fig. 5C. Lactobacillales was positively correlated with breast muscle b* and leg muscle water loss rate (%) and negatively correlated with breast muscle pH24h, as were leg muscle a* and leg muscle pH24h (*P* < 0.05, *P* < 0.01, *P* < 0.01, and *P* < 0.05, respectively), as illustrated in Fig. 5C. At the family level, Rikenellaceae was positively correlated with breast muscle L* and the water loss rate (%) but negatively correlated with breast muscle pH24h (*P* < 0.05 and *P* < 0.01, respectively), as depicted in Fig. 5D.

**Fig. 6:**
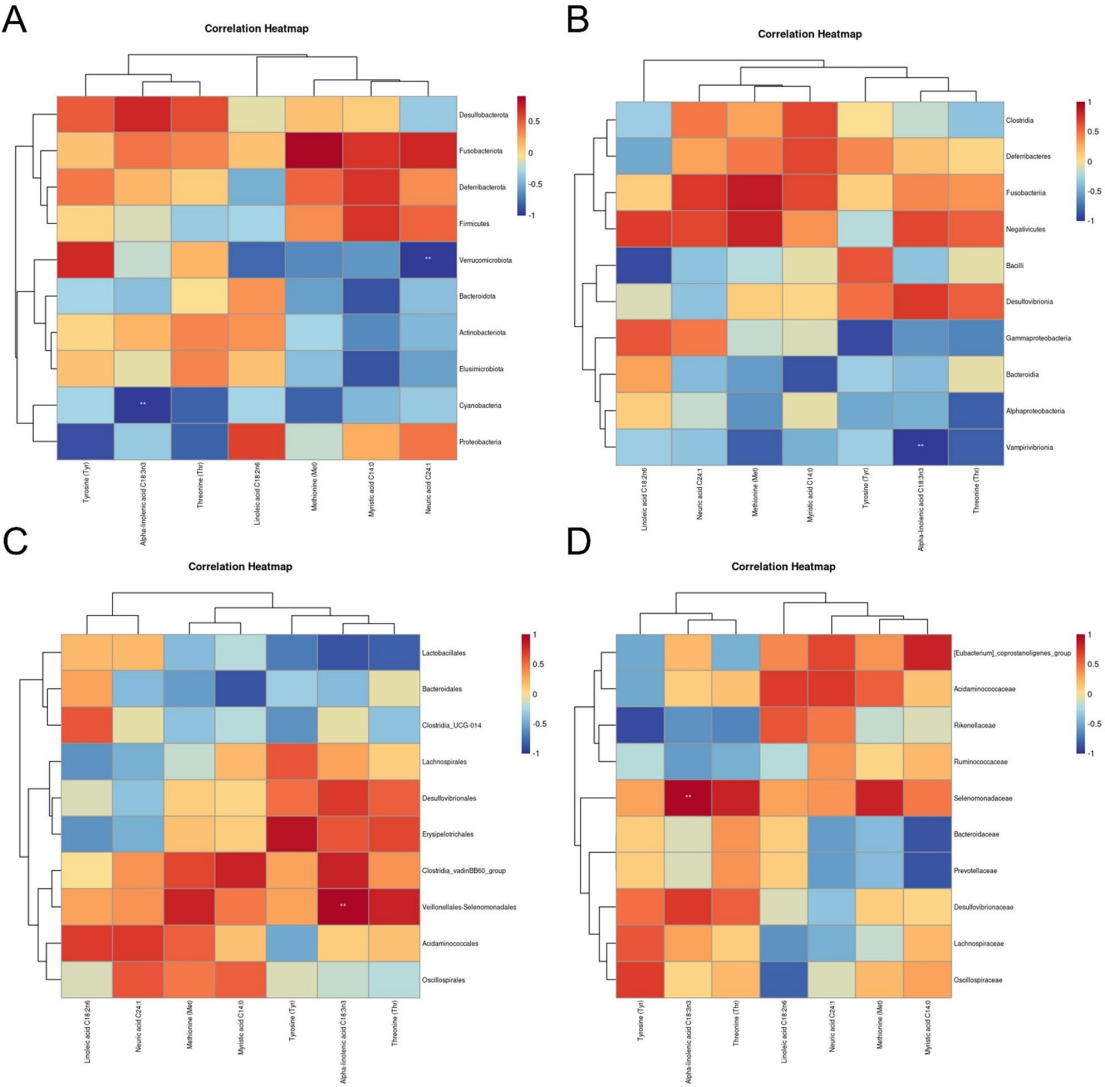
Spearman’s correlation analysis between the gut microbiota and the amino acid and fatty acid profiles of Leizhou black duck at the phylum (**A**), class (**B**), order (**C**) and family (**D**) levels. Spirillaceae abundance paralleled greater threonine/tyrosine and lower nervonic acid, reflecting microbiota-driven protein and lipid modulation

At the genus level, Spearman’s correlation analysis revealed significant inverse correlations between Verrucomicrobiota and nervonic acid C24:1 (*P* < 0.01) and negative correlations between Cyanobacteria and alpha-linolenic acid C18:3n3 (*P* < 0.01), as shown in Fig. 6A. At the class level, Vampirivibrionia was negatively correlated with alpha-linolenic acid C18:3n3 (*P* < 0.01), as presented in Fig. 6B. Order-level analysis revealed a positive correlation between Veillonellales-Selenomonadales and alpha-linolenic acid C18:3n3 (*P* < 0.01), as depicted in Fig. 6C. At the family level, Selenomonadaceae was positively correlated with alpha-linolenic acid C18:3n3 (*P* < 0.01), as shown in Fig. 6D.

## Discussion

### The impact of Rhodotorula mucilaginosa ZTHY2 on the growth performance of Leizhou black ducks

Rhodosaccharomyces colloides, which was rich in cellular proteins, small molecular peptides, vitamins, and unsaturated fatty acids, was easily absorbed and utilized by animals, directly participating in their metabolic processes [8]. It was commonly used as an animal nutritional supplement because of its palatable taste, which was enhanced by the interaction of flavor-releasing nucleotides and umami amino acids, stimulating animal appetite [3]. In this study, incorporating Rhodotorula mucilaginosa ZTHY2 into the duck diet significantly improved the average daily gain (ADG) and feed conversion ratio (F/G) in Leizhou black ducks, corroborating previous research [3]. The average daily feed intake (ADFI) of Leizhou black ducks significantly increased during the early growth phase (1–21 days), likely due to the immature thermoregulatory and digestive systems of the ducklings at this stage [20]. Introducing ZTHY2 into the duck feed promoted metabolism, improved the feed conversion rate, and provided additional energy and nutritional support for the growth and development of Leizhou black ducks via colonization and nutrients synthesis in the duck gastrointestinal tract. Therefore, this study revealed that dietary supplementation with Rhodotorula mucilaginosa ZTHY2 can substantially enhance the growth performance of Leizhou black ducks; at 2 × 10⁹ CFU/kg, strain ZTHY2 can replace growth-promoting antibiotics in local waterfowl such as Leizhou black ducks, enabling an antibiotic-free production system and markedly reducing feed costs and antibiotic-residue risks.

### The impact of Rhodotorula mucilaginosa ZTHY2 on the slaughter performance of Leizhou black ducks

The slaughter yield, complete evisceration rate, partial evisceration rate, pectoral muscle yield, and leg muscle yield were key indicators of livestock and poultry slaughter efficiency. They were crucial for evaluating the growth and nutritional efficiency of these animals, directly reflecting the economic returns from duck breeding [21]. Slaughter efficiency was a key metric for assessing duck meat production, with high-performing duck breeds usually showing a slaughter yield above 80% and an evisceration rate over 60% [22]. The current study revealed that Leizhou black ducks had a high slaughter yield of 85.22–88.62% and a complete evisceration rate of 72.80–76.13%, indicating their excellent meat production qualities. The addition of 2 × 10^9^ CFU/kg ZTHY2 to duck feed significantly improved the slaughter rate of these ducks. Sun Xifeng reported increased slaughter yield, pectoral muscle yield, and leg muscle yield in broilers as the dietary concentration of Lactobacillus plantarum JM113 increased [23]. Rhodiomyces vulvae, during its growth phase, accumulated nutrients and secreted active enzymes such as cellulase, amylase, protease, and lipase, which improved nutrient balance, promoted digestion and absorption, and increased slaughter efficiency.

### Influence of Rhodotorula mucilaginosa ZTHY2 on the muscle quality of Leizhou black ducks

The assessment of meat quality primarily involved crucial indicators such as meat color, pH level, and shear force, which were vital for determining the economic value of poultry meat and providing essential data for production performance analysis. The moisture content in poultry was commonly used to measure muscle moisture retention capacity, and a decrease in this capacity could lead to a loss of nutritional value and flavor. The water loss ratio and cook loss were the primary indicators of moisture content, with lower values indicating a higher moisture retention capacity within the muscular system [24]. Changes in muscle pH could lead to lactic acid build-up, causing muscle protein degeneration and a loss of water-binding capacity, reflecting the rate of lactic acid production from muscle glycogen fermentation and postmortem pH changes. Shear force was a measure of meat tenderness, and higher values indicate a firmer texture. As the meat pH decreases, both the moisture content and tenderness typically decreased [25]. Meat color was a vital indicator of muscle freshness and quality and was dependent primarily on the content and oxidation state of myoglobin within the muscle [26]. Meat color was commonly described via L* (luminance), a* (redness), and b* (yellowness) values. A higher L* value indicated a lighter meat color and poorer water quality, whereas higher a* values suggested less muscle oxidation and fresher meat [27]. Conversely, the b* value was directly proportional to the presence of sulfide myoglobin, with higher b* values correlating with poorer meat quality [25].

In this study, ZTHY2 administration significantly reduced the water loss ratio, pH_24h_, and L* and b* values of the breast and leg muscles of Leizhou black ducks while increasing the a* value in leg muscles at 2 × 10^9^ CFU/kg. The tenderness of the muscles also improved. This effect might have occurred due to the intracellular synthesis of red pigments, such as β-carotene and γ-carotene, in yeast cells, which were deposited in animal tissues, enhancing body and flesh color [28]. Additionally, under the influence of lycopene cyclase and β-carotene ketoenzymes, β-carotene within saccharomycetin cells catalyzed the production of astaxanthin and β-glucan, which were antioxidant compounds that boost antioxidant defenses in animals [29]. He et al. demonstrated that dietary supplementation with 200 mg/kg β-glucan from yeast could effectively increase the pH value and systemic water-holding capacity of growing pig muscle [30]. Therefore, ZTHY2 enhanced animal antioxidant capabilities by slowing glycogen anaerobic glycolysis and myoglobin oxidation, which increased the rate of pH decline, lowered the muscle moisture loss ratio, and contributed to the maintenance of muscle freshness and meat color consistency.

### Influence of Rhodotorula mucilaginosa ZTHY2 on the conventional nutritional components of the muscle of Leizhou black ducks

Determining the contents of conventional nutrients such as intramuscular crude protein and crude fat were pivotal in assessing meat quality and palatability, directly influencing the nutritional profile of muscle meat [27, 31]. Emerging research suggested that nutritional enhancement through the inclusion of feed additives, such as microbial agents, represented a viable strategy for increasing the nutritional value of animal muscle [32–34]. Our findings indicated that the integration of ZTHY2 into the duck diet could increase the crude protein content in both breast and leg muscles while reducing the crude fat content in breast muscles without substantially affecting moisture and ash contents. This effect might be attributed to the bioactive constituents, including lutein and astaxanthin, produced by Saccharomyces cerevisiae [32], which could modulate protein synthesis and fat metabolism pathways to reduce fat deposition, thereby increasing the crude protein content in the carcass. Hence, the effect of these variables on the interpretation of outcomes should be carefully considered during analysis.

### The regulating effects of Rhodotorula mucilaginosa ZTHY2 on the composition of amino acids and fatty acids in the muscle of Leizhou black ducks

The content and composition of amino acids and fatty acids were recognized indices for evaluating the nutritional value and flavor of meat. Sugars, amino acids and unsaturated fatty acids in muscle could directly or indirectly generate aldehydes, ketones, acids, alcohols, alkanes and heterocyclic compounds through the Maillard reaction and other intermediate products. At present, there were no reports on the effects of Saccharomyces cerevisiae on the composition of amino acids and fatty acids in duck muscle. However, in recent years, improving the composition of amino acids and fatty acids in meat products through nutritional strategies had also been shown to be feasible [31, 35]. Amino acids could be divided into four categories according to taste characteristics: umami amino acids, sweet amino acids, bitter amino acids and aromatic amino acids [36].

In this study, ZTHY2 significantly increased the accumulation of essential amino acids (EAAs), nonessential amino acids (NEAAs), and dipeptide amino acids (DAAs) in Leizhou black duck muscle tissue. Notably, when supplemented at a dosage of 2 × 10^9^ CFU/kg, ZTHY2 increased the content of flavor-enhancing amino acids, such as threonine and tyrosine. Tyrosine, an aromatic amino acid, improves meat quality and flavor because of its impact on the amino acid profile. As a functional amino acid, tyrosine plays a crucial role in animal metabolism, regulating physiological processes and supporting normal growth and development [37]. According to the FAO/WHO standard model, an ideal protein source should contain approximately 40% essential amino acids (EAAs) relative to total amino acids (TAAs), with more than 60% EAA compared with nonessential amino acids (NEAAs). On the basis of these experimental findings, Leizhou black duck was identified as a superior protein source, and the integration of ZTHY2 into the duck diet could further increase the nutritional value of duck meat and increase its high-quality protein content.

The fatty acid profile of meat significantly impacted both quality and health, with variety being key for assessing muscle lipid stability and meat product quality. The juiciness and flavor of muscle were closely related to fatty acid content. This study revealed that the main fatty acids in Leizhou black duck muscle were oleic, linoleic, palmitic, stearic, and arachidonic acids, which was consistent with findings from other meat duck breeds [38]. Research indicated that increased concentrations of saturated fatty acids (SFAs) and monounsaturated fatty acids (MUFAs) within muscle tissue were positively associated with increased tenderness, juiciness, and flavor, whereas polyunsaturated fatty acid (PUFA) content was inversely related to meat quality [17, 39]. The quality of meat was directly affected by lipid oxidation status, and polyunsaturated fatty acids (PUFAs) were more prone to oxidation than were saturated fatty acids (SFAs) and monounsaturated fatty acids (MUFAs). An increase in PUFA content accelerated the oxidation of fatty acids to myoglobin, leading to a reduction in muscle quality. Therefore, a moderate decrease in PUFA content could improve the flavor attributes of meat products.

In this study, ZTHY2 administration significantly reduced the proportion of nervonic acid in the muscle of Leizhou black ducks while increasing the ratios of MUFA and PUFA. Although the nervonic acid content decreased, there was a corresponding increase in the proportions of palmitoleic acid, oleic acid, and other beneficial fatty acids. In general, ZTHY2 probiotics had been shown to improve muscle fat profiles by reducing the proportion of polyunsaturated and saturated fatty acids while significantly increasing the levels of monounsaturated fatty acids, thus increasing meat quality and flavor without negatively impacting the fatty acid composition of Leizhou black duck muscle. Moreover, its ability to improve meat color, increase breast-muscle protein content, and decrease drip loss could directly raise dressing percentage and market price, offering farmers a measurable economic benefit.

### The regulating effect of Rhodotorula mucilaginosa ZTHY2 on the intestinal microbiota of Leizhou black ducks can be enhanced

The intestinal microbiota played a pivotal role in the intestinal ecosystem, with extensive research indicating its capacity to directly and indirectly modulate intestinal barrier functions and the assimilation of nutrients in animals by converting dietary components into bioactive metabolites [40, 41]. Administration of ZTHY2 significantly increased the Chao1 and Shannon indices of the cecum in Leizhou black ducks, suggested increased richness and diversity of the gut microbiota. However, the overall richness of the gut flora remained relatively unchanged. Beta diversity analysis revealed that ZTHY2, derived from Rhodosaccharomyces colulosa, regulated the composition of the intestinal microbiota, which was notably different from that in the control group. These findings supported previous research indicating that the relative abundance of specific bacterial groups may be altered by manipulating the gut microbiota composition without compromising overall alpha and beta diversity [5, 8].

The composition, relative abundance, and metabolic products of the intestinal microbiota exert multiple influenced on the host’s nutritional metabolism, the maturation of the intestinal immune system, and the maintenance of intestinal health. Additionally, these microorganisms actively engaged in the catabolism and homeostatic regulation of intestinal nutrients via their intrinsic biochemical responses and functionalities [33, 42]. Therefore, in this study, we analyzed alterations in the floral structure and biodiversity of the intestinal microbiota, revealing that Bacteroides dominated in Leizhou black ducks. The administration of ZTHY2 significantly increased the relative abundance of Actinobacteria and concurrently decreased the relative abundance of Proteobacteria, including those of the subclasses α-Proteobacteria and γ-Proteobacteria. Actinobacteria primarily preserved intestinal homeostasis by producing host-benefiting enzymes, bacteriostatic agents, immunomodulatory compounds, and bioactive metabolites [43]. Moreover, actinomycetes included a spectrum of beneficial bacteria, including species such as Bifidobacterium and Streptomyces, which performed physiological functions such as nutritional provision, bacteriostasis, and immune modulation, thereby playing a pivotal role in maintaining intestinal homeostasis [44, 45]. In contrast, the phylum Proteobacteria included various pathogenic and opportunistic organisms, such as Vibrio cholerae, Salmonella, Escherichia coli, and Helicobacter pylori, among others. An increased prevalence of Proteobacteria indicates a disrupted intestinal microbiota in hosts, with research revealing that animal immune system dysfunction correlates with excessive gut colonization by gamma-Proteobacteria, triggering chronic intestinal inflammation [46]. ZTHY2’s remarkable stress resilience likely underlied this phenomenon. Upon reaching the animal’s gastrointestinal tract, this yeast shifted to aerobic metabolism, competing with harmful bacteria for oxygen. This created a relatively anaerobic environment in the gut, which favored the growth of beneficial bacteria, such as lactic acid bacteria and Bifidobacterium, while effectively excluding pathogens. As a result, the composition and abundance of the intestinal microbiota in Leizhou black ducks were improved, preserving intestinal health and increasing disease resistance.

ZTHY2 administration notably increased the relative abundance of the Spinospiraceae family and significantly decreased the relative abundances of Clostridium UCG-014, Rienaceae, and coprosterogenes, including their respective subgenera. The increase in the relative abundance of Clostridium UCG-014 among proinflammatory bacteria could lead to the secretion of inflammatory cytokines and the upregulation of lipid proinflammatory metabolites [47]. Known for their ability to break down soluble polysaccharides and convert insoluble cellulose, Rienaceae also played a pivotal role in the intestinal tract. Additionally, fluttering Spirillaceae were adept at producing butyric acid and other short-chain fatty acids (SCFAs), which contributed to anti-inflammatory and antitumor effects [48]. The growth of Spirillaceae was closely related to a high concentration of short-chain fatty acids (SCFAs), which help maintain pH balance in the animal intestinal tract. A good pH balance inhibited the growth of harmful bacteria, promoted intestinal health, and improved feed efficiency [49]. The intestinal pH alteration, likely due to ZTHY2’s ability to ferment intestinal contents, involved the production of organic acids such as lactic, pyruvate, and acetic acids. This mechanism helped counter pathogen invasion by lowering the pH of the intestines [50]. The Saccharomyces colloides cell wall contained high levels of immunological polysaccharides such as β-glucan and mannooligosaccharides. These proteins binded competitively to the mannose-lectin receptors of pathogenic bacteria, prevented intestinal epithelial cell adherence and improved the gut microenvironment. This processed aids in epithelial cell differentiation and maturation, inhibited apoptosis, and enhanced nutrient and energy efficiency, promoting animal growth and development [51, 52].

Furthermore, through LEfSe analysis, distinct biomarkers linked to intestinal microbiota variability were identified in this study across different groups. These findings suggested that the dosage of ZTHY2 in the feed affected the gut microbiota of Leizhou black ducks, altering the relative abundance of key intestinal flora among the different treatment groups. Notably, key genera and species-level biomarkers, such as Rikenellaceae_RC9_gut_group, Lactobacillus, Streptococcus_alactolyticus, Lachnoclostridi Bacterium_phocaeense, Prevotellaceae_Ga6A1_group, Bacteroides_gallinaceum, Muribaculaceae, and Eubacterium_brachy_group, were identified to delineate taxonomic differences. Correlation analysis revealed significant relationships (*P* < 0.05 or *P* < 0.01) between these biomarker groups and factors such as ZTHY2’s influence on the intestinal flora, intestinal health, meat quality, and flavor of Leizhou black duck. However, further study was required to elucidate the impact of these biomarkers on Leizhou black duck intestinal development and immunity and on their potential to modulate metabolite expression. This research would undoubtedly enhance our understanding of intestinal microbiome mechanisms and host interactions.

In interpreting the present findings, we benchmarked our work against the literature across four dimensions—experimental design, sample size, analytical breadth, and practical relevance—thereby highlighting three key strengths: (i) the first duck-specific, dose–response assessment that concurrently employs a positive-bacterial control and an antibiotic-free basal diet, (ii) an integrated examination of meat-quality attributes and gut-microbiome dynamics, and (iii) immediate applicability to antibiotic-free production of local Leizhou black ducks. Conversely, the study was constrained by (a) the lack of serum immunological markers, (b) the absence of a high-temperature challenge, and (c) the exclusion of female birds, limitations that should guide future investigations.

We proposed that ZTHY2 could synthesize β-carotene and short-chain fatty acids that activated PPAR-γ in jejunal epithelia, thereby up-regulating claudin-1 and occludin, tightening intercellular junctions, reducing endotoxin flux and improving villus-height-to-crypt-depth ratio. The consequent enhancement of paracellular permeability increased amino-acid uptake and created a luminal environment that favored vitamin B12-synthesizing Spirillaceae while suppressing lipopolysaccharide-rich Fusobacteriaceae; both shifted lower local IL-6 and TNF-α levels, further sparing amino acids from catabolism and channeling them into pectoralis muscle protein accretion and water-holding capacity. Collectively, these molecular and microbial events provided a mechanistic explanation for why 2 × 10^9^ CFU kg⁻¹ ZTHY2 could fully replace antibiotic growth promoters without residues or loss of performance. To translate these findings into commercial practice, transcriptomic and metabolomic profiling coupled with large-scale field trials were next warranted to validate dose robustness across genotypes, production climates and feed manufacturing conditions.

## Conclusion

The present study demonstrated that dietary Rhodotorula mucilaginosa ZTHY2 supplied at 2 × 10⁹ CFU/kg achieved growth, meat-quality and gut-health benefits equivalent to, or better than, antibiotic-containing regimes without leaving residues. Consequently, this strain could be immediately recommended as a viable antibiotic substitute for Leizhou black ducks and potentially other waterfowl species. To consolidate these findings, multi-omics characterization and large-scale production trials should next be conducted to confirm its commercial value and regulatory suitability.

## Supplementary Material

Supplementary Material 1.

## Data Availability

The authors confirm that all the data used in the article supporting this study are available within the article. The datasets generated and/or analysed during the current study are available in the https://www.ncbi.nlm.nih.gov/bioproject/PRJNA1322124.

## Abbreviations

Abbreviations: Full Names
a*: Green-Red Component
ADFI: Average Daily Feed Intake
ADG: Average Daily Gain
ASV: Amplicon Sequence Variant
b*: Blue-Yellow Component
BW: Body Weight
CD: Crypt depth
CFU: Colony Forming units
EAA: Essential Amino Acids
FCR: Feed Conversion Ratio
L*: Lightness
LA: Lactobacillus Acidophilus
LDA: Linear Discriminant Analysis Effect Size
M: Model group
NEAA: Non-Essential Amino Acids
PCR: Polymerase Chain Reaction
pH: Potential hydrogen
RM: Rhodotorula mucilaginosa`
SV: Structural Variation
TFAA: Total Free Amino Acids
UAA: Umami Amino Acids
VH: Villus height
