# Supplementary material for: Rhodotorula mucilaginosa ZTHY2 as a promising antibiotic alternative in Leizhou black ducks: impact on growth, meat quality, intestinal health, and microbiota composition
Source: BMC Vet Res. 2026 Jan 30;22:130. doi: 10.1186/s12917-026-05292-3 (PMC12930758; doi:10.1186/s12917-026-05292-3)
Supplement: Supplementary file 1 — Supplementary Material 1. [file 12917_2026_5292_MOESM1_ESM.docx]

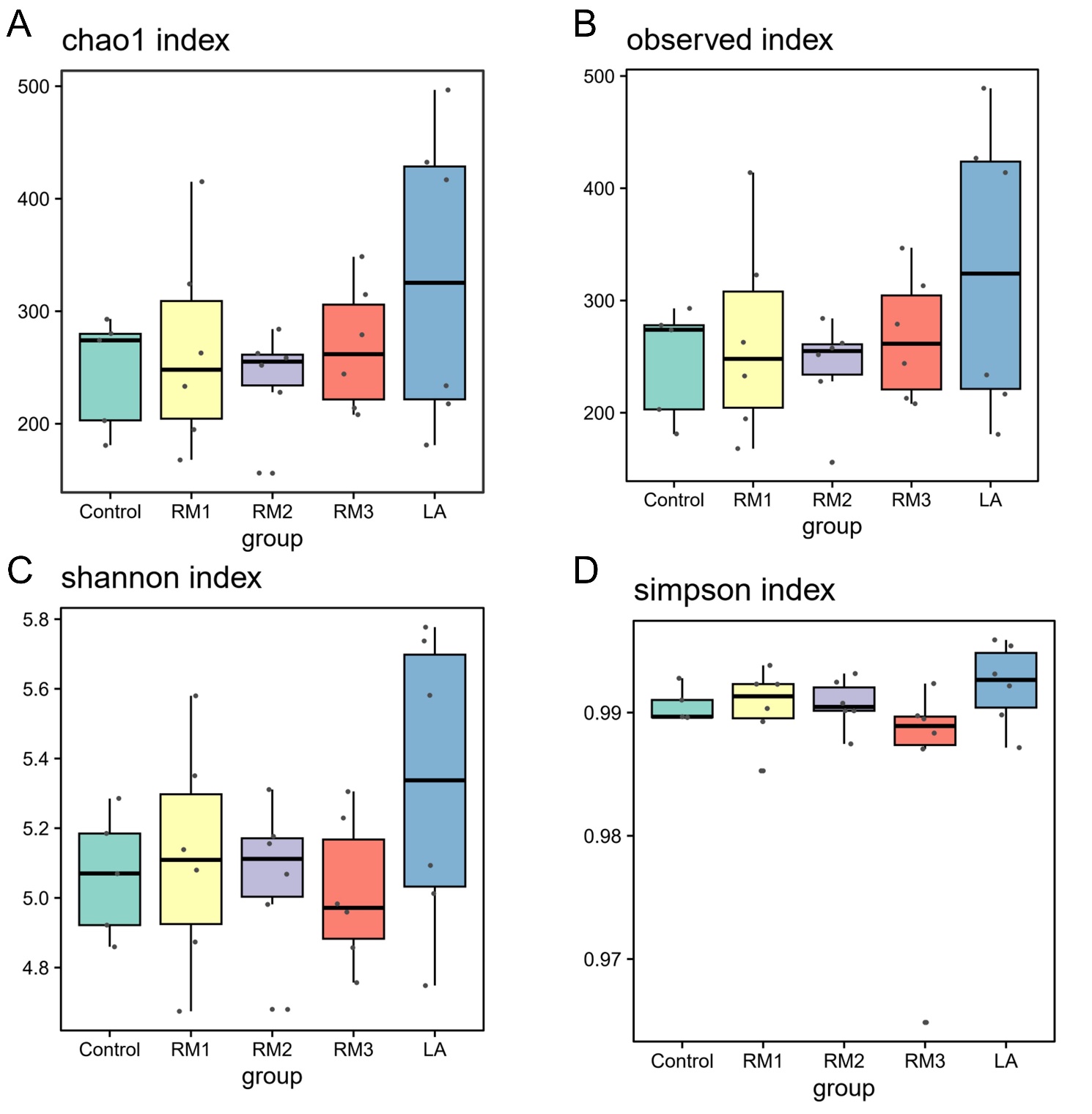


**Figure s1.** Alpha diversity analysis of the cecal microbiota in Leizhou black ducks. A Chao1 index; B. Observed species index; C. Shannon index; D. Simpson index. Control (Control group), RM1 (2 × 10^7^ CFU/kg RM group), RM2 (2 × 10^8^ CFU/kg RM group), RM3 (2×10^9^ CFU/kg RM group), LA (2×10^9^ CFU/kg LA group). RM, Rhodotorula mucilaginosa; LA, Lactobacillus acidophilus.


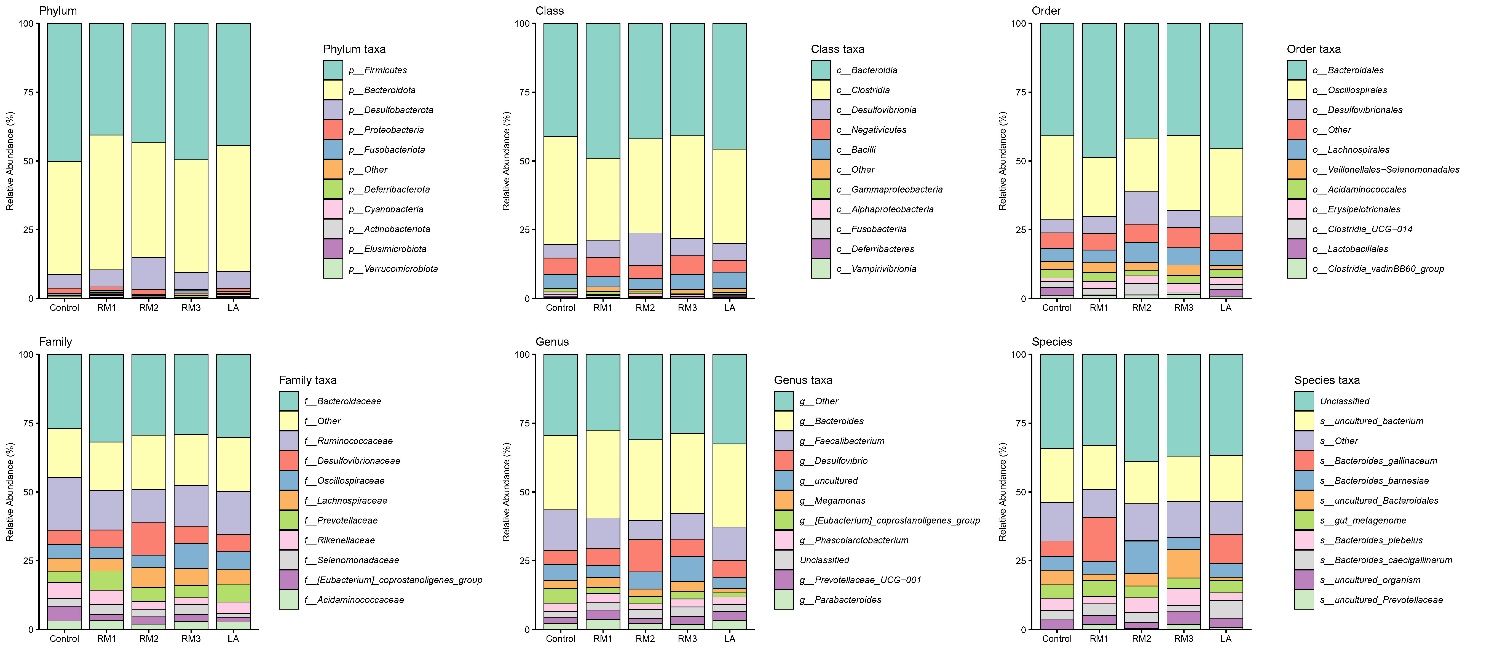


**Figure s2.** The structure and abundance of microorganisms at the phylum, class, order, family, genus and species levels in Leizhou black ducks. Control (Control group), RM1 (2 × 10^7^ CFU/kg RM group), RM2 (2 × 10^8^ CFU/kg RM group), RM3 (2×10^9^ CFU/kg RM group), LA (2×10^9^ CFU/kg LA group). RM, Rhodotorula mucilaginosa; LA, Lactobacillus acidophilus.


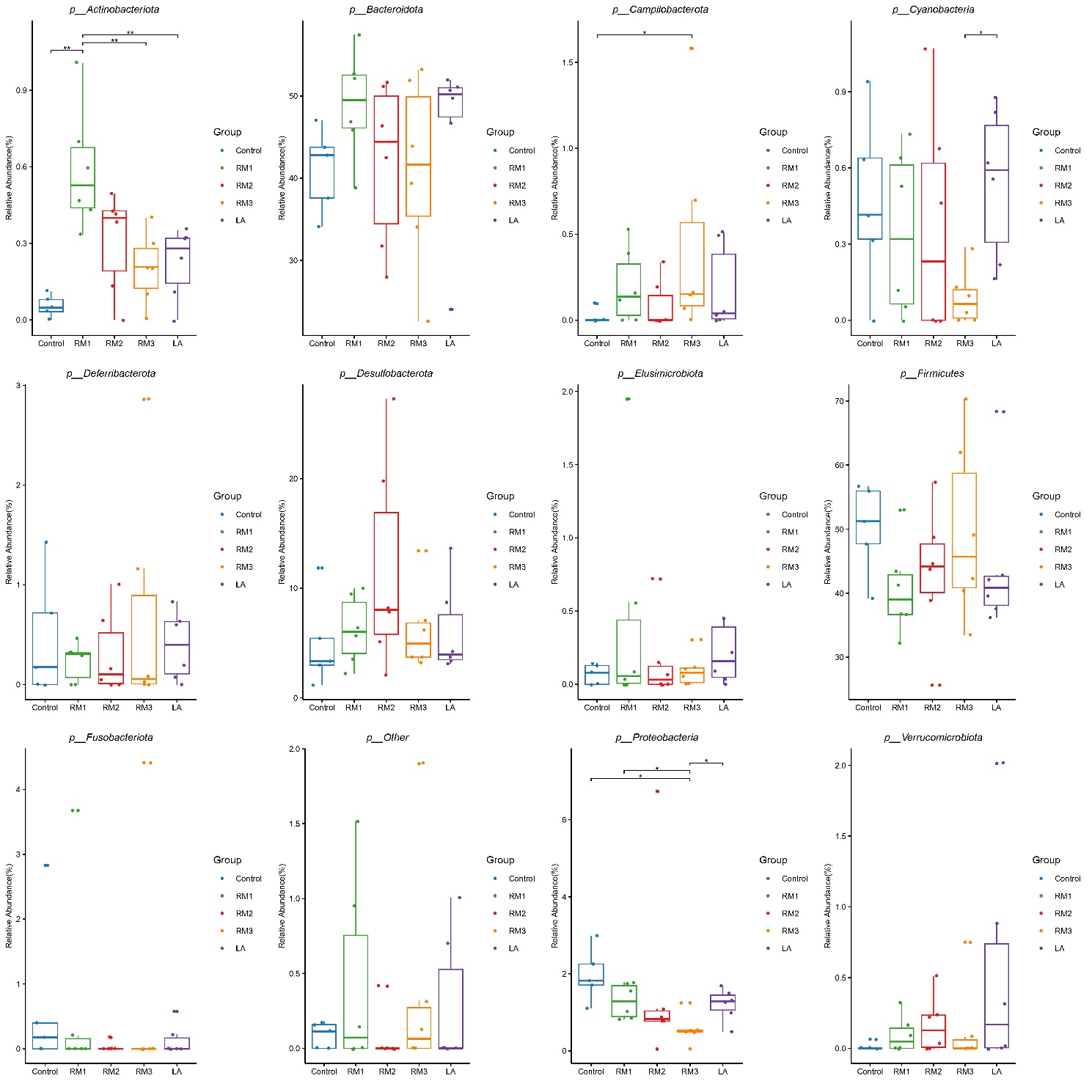


**Figure s3.** Abundance of the cecal microbiota at the phylum level in Leizhou black ducks. Control (Control group), RM1 (2 × 10^7^ CFU/kg RM group), RM2 (2 × 10^8^ CFU/kg RM group), RM3 (2×10^9^ CFU/kg RM group), LA (2×10^9^ CFU/kg LA group). RM, Rhodotorula mucilaginosa; LA, Lactobacillus acidophilus.


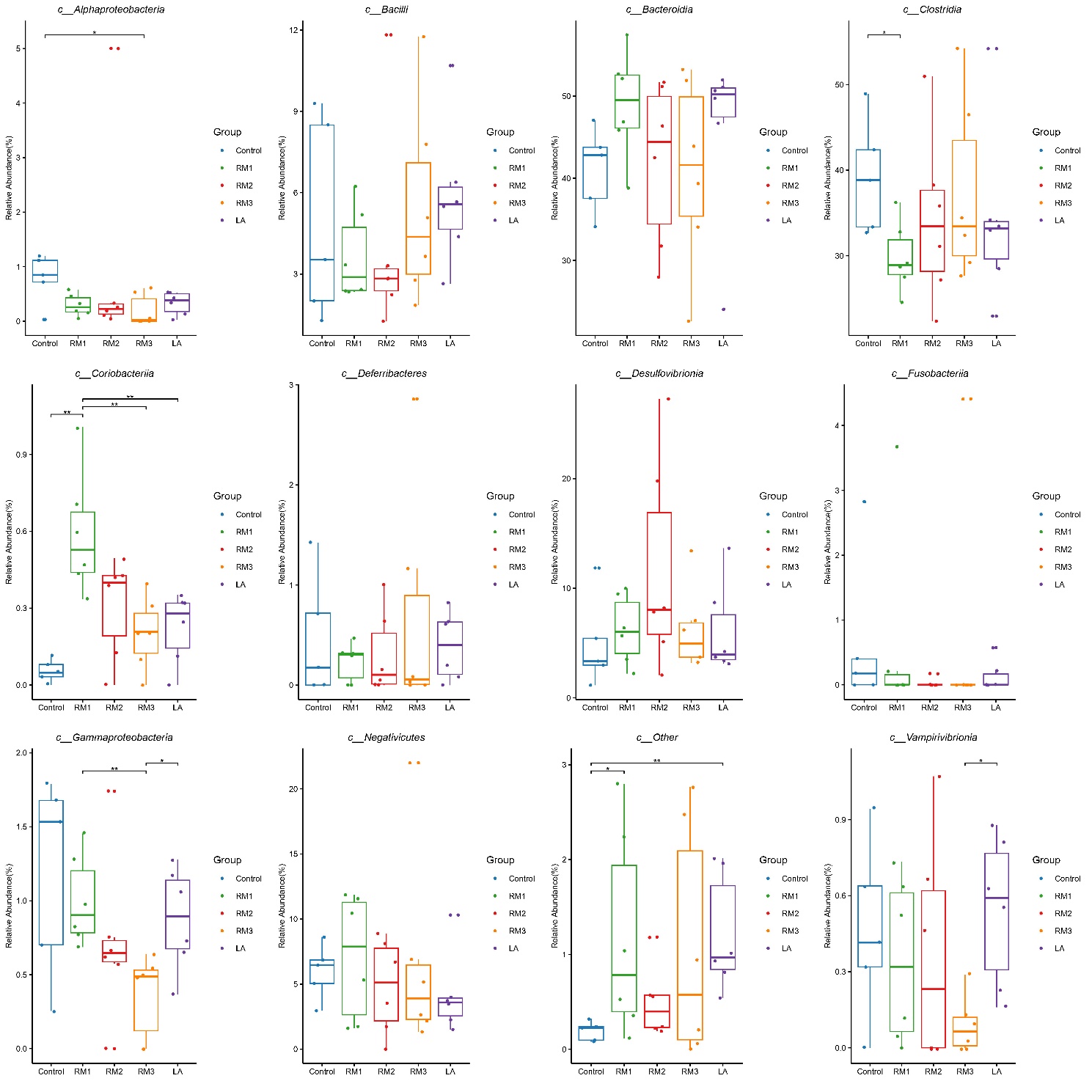


**Figure s4.** Abundance of the cecal microbiota at the class level in Leizhou black ducks. Control (Control group), RM1 (2 × 10^7^ CFU/kg RM group), RM2 (2 × 10^8^ CFU/kg RM group), RM3 (2×10^9^ CFU/kg RM group), LA (2×10^9^ CFU/kg LA group). RM, Rhodotorula mucilaginosa; LA, Lactobacillus acidophilus.


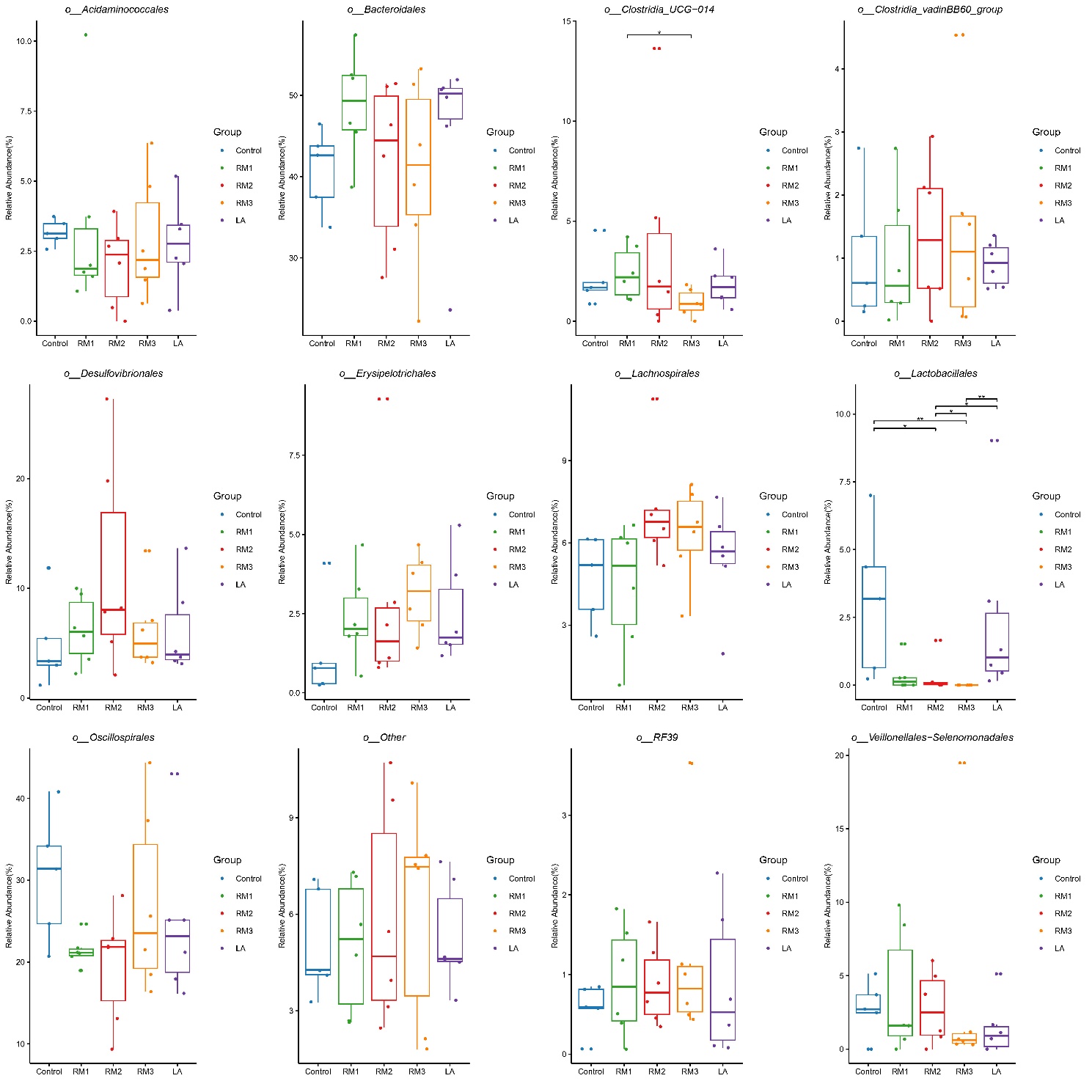


**Figure s5.** Abundance of the cecal microbiota at the order level in Leizhou black ducks. Control (Control group), RM1 (2 × 10^7^ CFU/kg RM group), RM2 (2 × 10^8^ CFU/kg RM group), RM3 (2×10^9^ CFU/kg RM group), LA (2×10^9^ CFU/kg LA group). RM, Rhodotorula mucilaginosa; LA, Lactobacillus acidophilus.


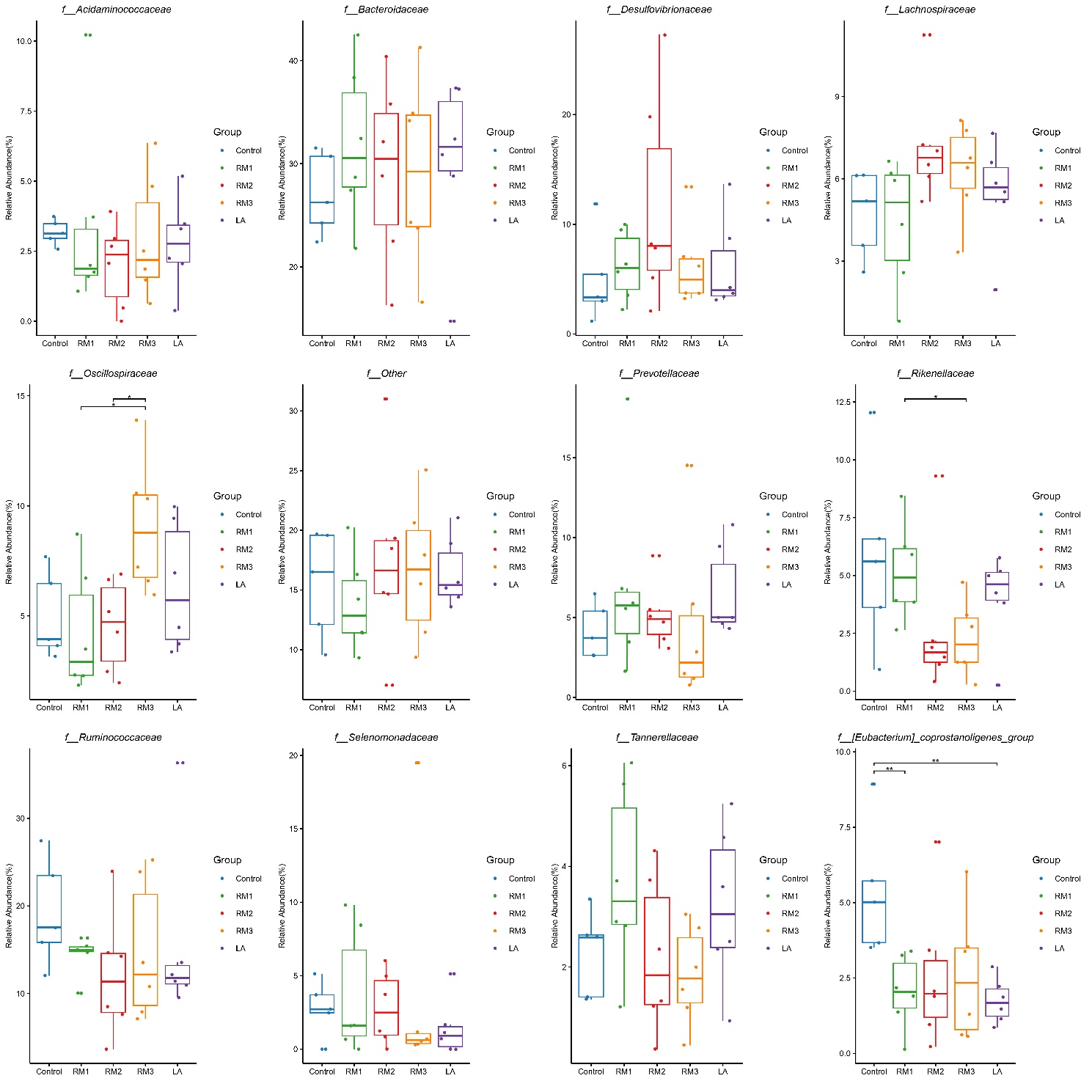


**Figure s6.** The abundance of the cecal microbiota at the family level in Leizhou black ducks. Control (Control group), RM1 (2 × 10^7^ CFU/kg RM group), RM2 (2 × 10^8^ CFU/kg RM group), RM3 (2×10^9^ CFU/kg RM group), LA (2×10^9^ CFU/kg LA group). RM, Rhodotorula mucilaginosa; LA, Lactobacillus acidophilus.


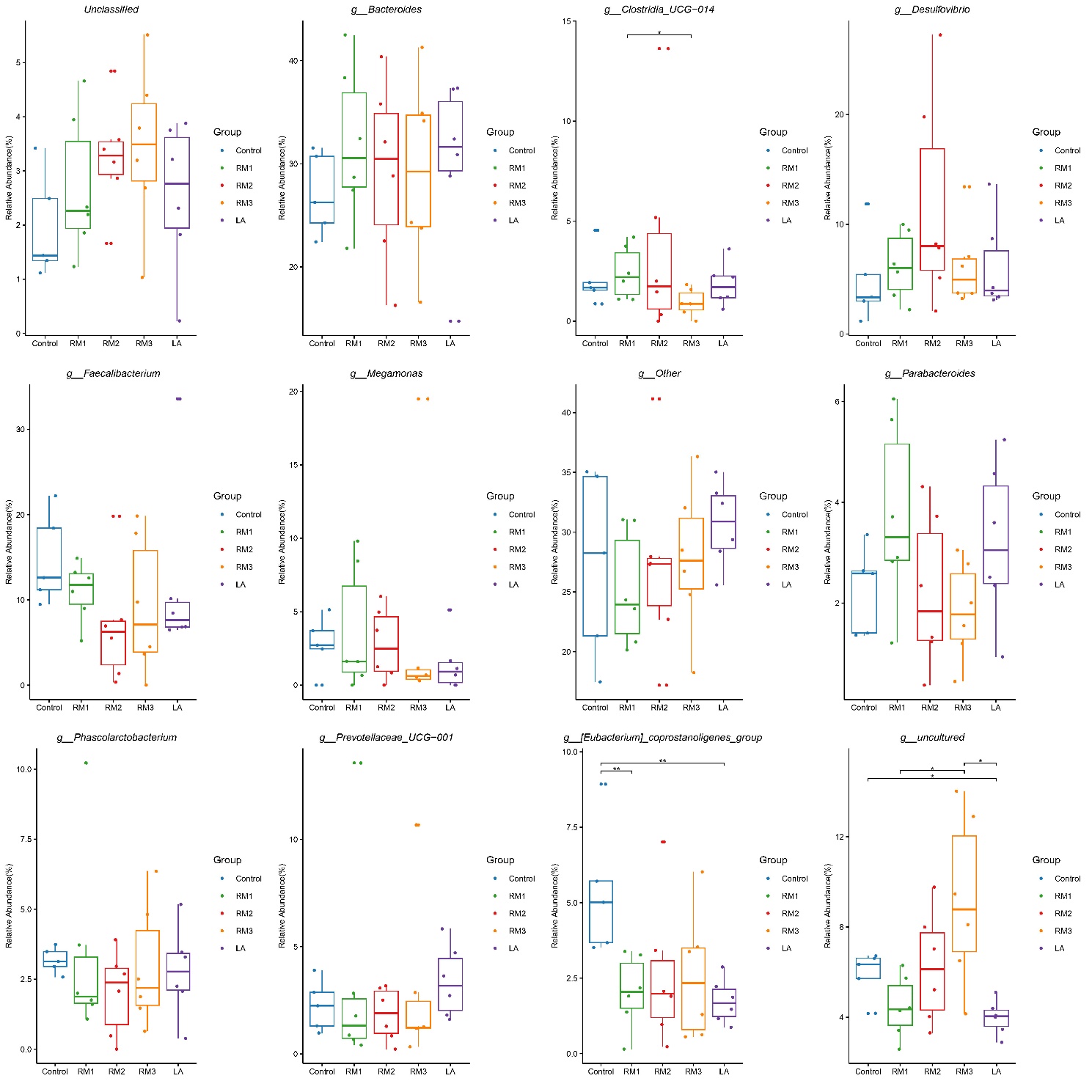


**Figure s7.** Abundance of the cecal microbiota at the genus level in Leizhou black ducks. Control (Control group), RM1 (2 × 10^7^ CFU/kg RM group), RM2 (2 × 10^8^ CFU/kg RM group), RM3 (2×10^9^ CFU/kg RM group), LA (2×10^9^ CFU/kg LA group). RM, Rhodotorula mucilaginosa; LA, Lactobacillus acidophilus.
